# Long-term stability and computational analysis of migration patterns of L-*MYC* immortalized neural stem cells in the brain

**DOI:** 10.1371/journal.pone.0199967

**Published:** 2018-08-02

**Authors:** Russell C. Rockne, Vikram Adhikarla, Lusine Tsaturyan, Zhongqi Li, Meher B. Masihi, Karen S. Aboody, Michael E. Barish, Margarita Gutova

**Affiliations:** 1 Department of Information Sciences, Division of Mathematical Oncology, Beckman Research Institute of City of Hope, Duarte, California, United States of America; 2 Department of Developmental and Stem Cell Biology, Beckman Research Institute of City of Hope, Duarte, California, United States of America; Universidad de Jaen, SPAIN

## Abstract

**Background:**

Preclinical studies indicate that neural stem cells (NSCs) can limit or reverse central nervous system (CNS) damage through delivery of therapeutic agents for cell regeneration. Clinical translation of cell-based therapies raises concerns about long-term stability, differentiation and fate, and absence of tumorigenicity of these cells, as well as manufacturing time required to produce therapeutic cells in quantities sufficient for clinical use. Allogeneic NSC lines are in growing demand due to challenges inherent in using autologous stem cells, including production costs that limit availability to patients.

**Methods/Principal findings:**

We demonstrate the long-term stability of L-*MYC* immortalized human NSCs (LM-NSC008) cells *in vivo*, including engraftment, migration, and absence of tumorigenicity in mouse brains for up to nine months. We also examined the distributions of engrafted LM-NSC008 cells within brain, and present computational techniques to analyze NSC migration characteristics in relation to intrinsic brain structures.

**Conclusions/Significance:**

This computational analysis of NSC distributions following implantation provides proof-of-concept for the development of computational models that can be used clinically to predict NSC migration paths in patients. Previously, models of preferential migration of malignant tumor cells along white matter tracts have been used to predict their final distributions. We suggest that quantitative measures of tissue orientation and white matter tracts determined from MR images can be used in a diffusion tensor imaging tractography-like approach to describe the most likely migration routes and final distributions of NSCs administered in a clinical setting. Such a model could be very useful in choosing the optimal anatomical locations for NSC administration to patients to achieve maximum therapeutic effects.

## Introduction

Stem cell-based therapies for tumors and neurodegenerative diseases depend on efficient delivery of stem cells to the areas of damage. Neural stem cells (NSCs) are inherently pathotropic and suggested to migrate to sites of tumor and damage in the central nervous system (CNS) [1]. Therefore, NSCs can be exploited for cell replacement, regeneration, and therapeutic delivery strategies. NSCs are currently being evaluated in clinical trials for stroke, multiple sclerosis, amyotrophic lateral sclerosis, Parkinson’s disease, and other CNS diseases [2–6]. In addition, we have previously shown that a genetically modified NSC line (HB1.F3.CD) can deliver anti-cancer agents selectively to invasive brain tumor sites [7–10]. Allogeneic NSC lines are in growing demand due to the limitations of adult autologous patient-derived NSCs. Although self-renewing NSCs are present in developing brain tissue, *in vitro* passages of these cells lead to decreased capacity for cellular self-renewal, decreased differentiation potential, and increased accumulation of chromosomal and functional instabilities [11–14]. Recently, we developed a new human NSC line, LM-NSC008, that was genetically engineered to stably express the L-*MYC* gene. This line has favorable physiological, multi-lineage differentiation and migratory properties [14]. The LM-NSC008 cell line overcomes several major hurdles to clinical translation of cell-based therapies by demonstrating long-term stability, lack of tumorigenicity, and ease of production in sufficient quantities for clinical trials. Further supporting this line’s potential for use in clinical applications, LM-NSC008 cells demonstrate tropism to sites of brain tumors and/or injury when administered intranasally or intracranially to the mouse brain [14]. Ultimately, characterization of the long-term stability and migratory properties of allogeneic LM-NSC008 cells will have a relevance for use of this cell line for proof-of-concept studies and may pave the way for successful translation of these cells to target neurodegenerative diseases.

Clinical translation of NSC-based therapy has been hampered by inability to quantify or predict NSC migration to sites of tumor or injury. A method for predicting NSC migration is needed because the migration route as well as the location of tumor/injury may affect the final number of viable cells that reach the tumor/injury site. We expect the characterization of these migration paths and quantification of the NSC viability at the tumor/injury site will ultimately allow for disease or injury site-specific modification of NSC dose and route of administration. We have recently published manuscripts describing use of 3-dimensional reconstructions to determine the distribution and coverage of brain tumors by therapeutic NSCs in orthotopic xenograft models of glioma [15, 16]. We have now used computational analyses of tissue structure, orientation, and anisotropy to characterize preferential routes of NSC migration, which may provide the ability to predict routes of migration and spatial distribution of LM-NSC008 cells within the brain. Successful development of a computational predictive model of LM-NSC008 migration in the brain would provide a quantitative method for analysis of repeat treatments for LM-NSC008 cells or other NSC lines, which could change the NSC treatment administration paradigm for patients with brain tumors or injuries.

## Materials and methods

### Cell culture

LM-NSC008 cells, stably expressing the *L-MYC* gene, were previously generated and characterized [14]. LM-NSC008 cells were cultured in serum-free NSC medium (RHB-A medium; Cell Science) supplemented with 10 ng/mL basic FGF (bFGF), 10 ng/mL EGF, 2 mM L-glutamine (Invitrogen), Gem21 NeuroPlex Serum-Free Supplement (GeminiBio-Products, #400–160), and penicillin- streptomycin (Mediatech, 30-002-CI) as previously described [14]. IncuCyte live cell analysis imaging system demonstrated similar growth kinetics of LM-NSC008 cells at p5 and p45 passages. LM-NSC008 cells were plated on 24-well plates at a density of 2 x 10^4^ cells/cm^2^ (40,000 cells/well). Cells were grown for 10 days at 37 ^0^C, media (RHB-A) change was performed every 3 days and imaging of eGFP–labeled LM-NSC008 cells was acquired every 12 h using the IncuCyte S3 Live Cell Analysis System (Sartorius). Experimental data is represented as the mean ± SD of 2 independent assays performed in quadruplicate.

### Genomic DNA and qRT-PCR analysis

LM-NSC008 cells were cultured and passaged in complete growth medium and harvested every 5th passage. Genomic DNA was isolated using the DNeasy kit (Qiagen) and PCR was performed to amplify the L-*MYC* gene. The PCR product was analyzed by electrophoresis on a 0.8% agarose gel and visualized by ethidium bromide staining. The L-myc-pMXs plasmid was used as a positive control and genomic DNA from untransduced NSC008 cells and no DNA template were used as the two negative controls. Total RNA was isolated using TRIzol LS reagent (Invitrogen) according to the manufacturer’s protocol. RNA (100 ng) was reverse transcribed into cDNA using the iScript cDNA Synthesis Kit (Bio-Rad). Quantitative real-time PCR (qRT-PCR) was performed in 25 μl reactions using the iQ SYBR Green Supermix Kit (Bio-Rad) and *L-MYC*-specific primers (L-*MYC*: 5'AGAGGCAGTCTCTGGGTATT3'; 5'TGTGCTGATGGATGGAGATG3') on a Bio-Rad CFX96. Values obtained from qRT-PCR were normalized to *GAPDH*, and the relative expression of the L-*MYC* gene was calculated using the 2^-ΔΔCt^ method. The intensities of the L-*MYC* PCR bands were analyzed using ImageJ software, quantified, and represented as percentage of the total size of measured area.

### Detection of expressed and secreted proteins

Protein array analysis was conducted using Cytokine Antibody Array V (RayBiotech, http://www.raybiotech.com). LM-NSC008 cells (p5 and p45) were cultured under conditions described previously [14] for 7 days. Culture media (CM) was collected and cells were pelleted by centrifugation at 16000 g and lysed with lysis buffer (20mM TRIS, 150 mM NaCl, 2 mM EDTA, 0.5% Triton X-100). Antibody array membranes were exposed to 1 mL of CM and 1 mL of cell lysates, containing 500 μg of total protein in blocking buffer. Membranes were incubated at 4°C overnight and chemiluminescence films were developed as per the manufacturer’s recommendations.

### *In vivo* animal studies

All animal studies were performed under an approved City of Hope Institutional Animal Care and Use Committee protocol (IACUC #12025). Male and female NOD scid IL2Rgamma null (NSG) mice (8–12 weeks old) were intracranially injected with 5 x 10^5^ NSCs/4 μl of PBS per mouse (n = 10) into the right frontal lobe. Six (Group 1, n = 5 mice) and nine months (group 2, n = 5 mice) after NSC injection, mice were euthanized and their brains isolated and post fixed in 4% paraformaldehyde (PFA). Mice developed no symptoms that required euthanasia. However, mice were monitored daily for the standard AAALAC criteria for euthanasia (e.g., 20% weight loss, domed head, ataxia) as well as any distress or discomfort in accordance with the recommendations of the Panel of Euthanasia of the American Veterinary Medical Association. Mice were euthanized by CO_2_ inhalation, with a gradual increase in the flow of CO_2_, allowing for observation of mice during euthanasia and minimizing distress.

After fixation, brains were rinsed with 1x PBS and transferred to 70% EtOH for dehydration (for 3–5 days) and later embedded into paraffin blocks (four mice per Groups 1 and 2). The paraffin blocks were sectioned into 10 μm sections and stained (every 10th section) with anti-human Nestin antibody (Millipore; Cat #: MAB5326) and DiI lipophilic tracer (Invitrogen; D282). Two brains (one from each Groups 1 and 2) were fresh frozen and immersed into Optimal Cutting Temperature Compound (OCT) over dry ice to be cryosectioned into 10 μm sections for staining with Stem123 antibody specific for human GFAP (Cellartis; Cat #: Y40420).

### IHC and DiI staining

Paraffin-embedded brain sections were deparaffinized in xylene and rehydrated with ethanol [17]. The brain sections were then processed for antigen retrieval with Proteinase K (Dako ready-to-use Cat #: S3020). The tissue sections were incubated in peroxidase quenching solution (0.3% H_2_O_2_ made in 100% methanol) for 20 min at room temperature and then in blocking solution for 1h at room temperature (50% BlockAid, Invitrogen B10710; 50% Western Blocking Reagent, Roche Applied Sciences 11921673001; 1% Triton-100x). Sections were then stained with primary antibody in blocking solution and incubated overnight at 4°C followed by four washes in PBS and reaction with biotinylated secondary antibody for 1 h as described previously (1:250 dilution, Vector BA-2001)[17]. Sections were washed in PBS, incubated in avidin-biotin complex (ABC) solution for 1 h at room temperature and 5 min in 3, 3′-Diaminobenzidine (DAB) substrate solution containing 0.25% H_2_O_2_. Then the brain sections were washed in PBS and mounted with Cytoseal 8 mounting media for bright field imaging (Richard-Allan Scientific). Staining with the Stem123 antibody followed the same protocol but on frozen sections fixed with 4% PFA. Stem123 primary antibody (Celartis, Cat #: Y40420) was used at a 1:1000 dilution and a secondary biotinylated antibody at 1:250 (Biotinylated Anti-Mouse IgG (H+L); Vector, Cat #: BA-2001). For DiI staining, the OCT-immersed brain sections were fixed with 4% PFA, dehydrated through a graded ethanol series, and stained with DiI lipophilic tracer for 15 min (DiIC18(3); Life Technologies; Cat #: D282). Fluorescent staining for HNA, Ki-67, hNestin, Pax6 and myelin basic protein (MBP) was performed using frozen brain sections and the same protocol. Primary antibodies were added in blocking solution and incubated with sections overnight at 4°C. Dilutions of the primary antibodies were as follows: HNA, 1:200 (Abcam, Cat #: ab191181); Ki-67, 1:100 (DAKO, Cat #: M7240); hNestin, 1:200 (Mouse Monoclonal IgG1 Anti-hNestin, EMD Millipore: Cat #: MAB5326); Pax6, 1:200 (Biolegend INC; Cat #: 901301) and myelin basic protein (MBP), 1:100 (Santa-Cruz Bio, Cat #: sc-271524). Slides were washed in PBS and incubated with secondary antibody (Goat Anti-Mouse IgG at 1:100, Jackson ImmunoResearch, Cat #: 115-587-003 or Goat Anti-Rabbit IgG, 1:100, Molecular Probes, Cat #: A11037) and DAPI solution for 1 h at room temperature. Slides were rinsed in PBS and mounted in fluorescence mounting media (DAKO, Cat #: S3023).

### Computational analysis of tissue anisotropy

Tissue anisotropy and orientation were quantified in DiI-stained *ex vivo* tissue using the OrientationJ plugin for FIJI [18] following methods outlined by Budde [19–21]. OrientationJ uses Fourier transform analysis to compute eigenvalues and eigenvectors of a structure tensor at each pixel in the DiI image. The eigenvalues (λ) are combined to produce measures of anisotropy (coherence), directionality (orientation), and other quantities that were not used in this analysis. Coherence (*C* = (*λ_max_* – *λ_min_*)/(*λ_max_* + *λ_min_*)) measures the degree to which there is a dominant eigenvalue of the structure tensor (see S2 Fig for illustration and discussion of these concepts). Coherence takes values from 0–1, with a value of 0 corresponding to isotropic and a value of 1 to anisotropic. Orientation is the angle of the dominant eigenvector measured from the positive abscissa axis. To generate the orientation and coherence maps, a cubic spline gradient method with a Gaussian window of 1 pixel standard deviation was used in the OrientationJ Analysis module of the OrientationJ plugin. The orientation of the white matter (WM) was compared to the orientation of NSCs with respect to each other. To calculate the orientation of NSCs the following procedure was used. The center of each NSC cluster defined by the nestin- or Stem123-stained image was dilated by 200 pixels and eroded by 100 pixels to create coalesced regions of NSC clusters yielding an NSC density map. A second degree polynomial was fit to each of these coalesced regions and a line tangent to this polynomial and closest to each NSC cluster center was calculated. The angle of this line with the abscissa indicates the orientation of NSCs (θ_NSC_) for that cluster.

## Results

### Characterization of LM-NSC008 cells *in vitro* and *in vivo*

**Fig 1:**
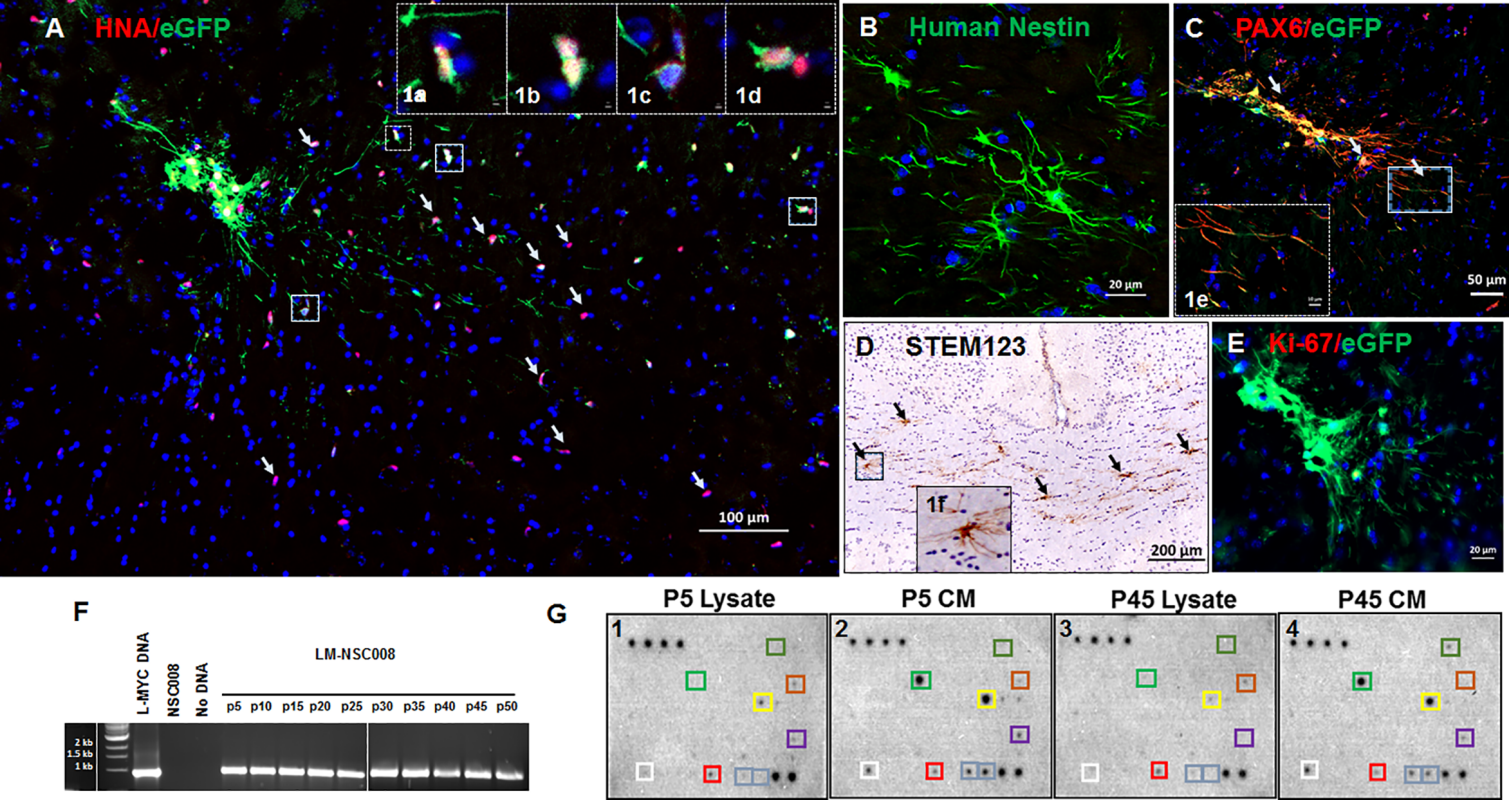
Characterization of LM-NSC008 cells. Visualization of LM-NSC008 cells in naïve non-tumor bearing mouse brain 6 and 9 months post administration. **(A)** Immunohistochemistry (IHC) staining of LM-NSC008 cells (stably expressing eGFP/Ffluc) with anti-HNA antibodies (NSC injection site, white arrows indicate the LM-NSC008 cells, scale bar 100 μm). **(1a-1d)** Insets show LM-NSC008 cells co-expressing HNA and eGFP proteins (inset scale bars [1a-1f] 2 μm). **(B)** IHC staining for huNestin (green) in mouse brain sections from 6 months after administration, scale bar 20 μm; **(C)** IHC staining for Pax6 (red)/eGFP in LM-NSC008 cells, scale bar 50 μm. Inset **(1e**) shows enlarged area of neuronal cells co-expressing Pax6 and eGFP. **(D)** Bright-field image of LM-NSC008 cells stained with Stem123 and contrastained with hematoxylin, scale bar 200 μm. Inset **(1f)** enlarged LM-NSC008 cell, expressing the glial marker Stem123. Arrows (black) show LM-NSC008s (brown) in the corpus callosum. **(E)** IHC for Ki-67, showing negative staining (red) of LM-NSC008 cells expressing eGFP (injection site), scale bar 20 μm. Fluorescent-stained slides were counterstained with DAPI (blue) to visualize nuclei. **(F)** PCR analysis of genomic DNA derived from LM-NSC008 cells at every 5th passage *in vitro* (p5-p50). Controls were: L-*MYC* plasmid, DNA derived from untransduced NSC008 cells, no DNA template. (**G**) Protein expression profiles from cell lysates (G1 and G3) and CM (G2 and G4) from LM-NSC008 cells at passages 5 and 45. Protein array analysis was conducted using Cytokine Antibody Array V from RayBiotech.

Characterization of LM-NSC008 cells was conducted in naïve non-tumor bearing mouse brain 6 or 9 months post NSC administration (Fig 1). To observe the behavior and migration of intracranially-injected LM-NSC008 NSCs, we intracranially injected LM-NSC008 cells, lentivirally transduced to express enhanced green fluorescent protein (eGFP) and firefly luciferase genes (Ffluc) (5 x 10^5^ cells/4 μl PBS) into the right frontal hemisphere of adult male and female NSG mice (n = 10). Six or nine months after NSC injection, mice were euthanized and their brains were harvested, fixed, sectioned, and stained for histological examination and immunohistochemistry (IHC) analysis. Human nuclear antigen (HNA) and eGFP protein expressions were used to visualize LM-NSC008 cells that migrated from the injection site in the mouse brain (Fig 1A). Analysis of co-expression of eGFP and HNA confirmed the human origin of LM-NSC008 cells in the mouse brain (Fig 1, insets 1a-1d). We also demonstrated that LM-NSC008 cells expressed other neuroectodermal-specific markers such as nestin and Pax6 at 6 and 9 months post administration (Fig 1B), implicating in the radial growth of axons (huNestin, green) (Fig 1B and 1C). Pax6 is uniformly expressed in the early neuroectoderm of human fetuses and in neural progenitors differentiated from hESCs and induced pluripotent stem cells (iPSCs). The LM-NSC008.eGFP cells (white arrows) were double positive for Pax6 (red)/eGFP (green), indicating multipotent differentiation of LM-NSC008 cells towards the neuronal lineage in non-tumor bearing naïve mouse brain at 9 months post administration (Fig 1C). Next, we performed IHC for the human-specific glial marker (Stem123), visualizing LM-NSC008 cells in the corpus callosum (Fig 1D). Ki-67 staining of mouse brain sections obtained at 9 months post administration indicated no expression of Ki-67 protein, suggesting that LM-NSC008 cells most likely were not dividing at the 9 month timepoint.

For *in vitro* studies, LM-NSC008 cells were grown and passaged up to 50 times under hypoxic conditions (4% O_2_) in a humidified incubator, as described previously [14]. In growth factor-supplemented stem cell medium, LM-NSC008 cells grew as a monolayer and showed no changes in growth rate up to 50 passages *in vitro* (S1A–S1C Fig). The continued presence of L-*MYC* in LM-NSC008 cells was confirmed by PCR analysis of genomic DNA at every fifth passage (p5–p50) (Fig 1F). L-*MYC* band intensities for every fifth passage of LM-NSC008 cells (p5 to p50) were compared using one-way ANOVA and showed no statistical difference among the bands when analyzed using the same sized area (P = 0.058). We also analyzed L-*MYC* gene expression at every fifth passage using real time PCR (RT-PCR) (data not shown). Our results indicate L-*MYC* gene expression is relatively stable in LM-NSC008 cells over the long-term *in vitro* passaging.

To assess protein secretion and expression by LM-NSC008 cells over passages *in vitro*, we performed protein array analyses using conditioned medium (CM, p5 and p45) and cell lysates (derived from pelleted cells, p5 and p45). LM-NSC008 cells from low (p5) and high (p45) passages were cultured for 5 days, after which CM was collected and cells were collected and pelleted. CM derived from low (p5) and high (p45) passages showed comparable cytokine secretion profiles (MCP-1, EGF, TIMP-1, TGF-beta 2, OPN, and IGFBP-2) (Fig 1G2 and 1G4). Similar results were observed in lysates derived from LM-NSC008 cells from low and high passages, suggesting the stability of protein expression *in vitro* (Fig 1G1 and 1G3).

We also demonstrated self-renewal and growth kinetics of LM-NSC008 cells at p5 and P45 *in vitro* (S1D Fig) using IncuCyte. Scale-up expansion of LM-NSC008 cells to a large cell banks (expansion of LM-NSC008 cells from 5 x 10^7^ cells to 3 x 10^9^ cells within 10 days) was demonstrated using a Quantum Cell Expansion System (S1E–S1G Fig)[22]. Collectively, we demonstrated *in vivo* migration and fate of LM-NSC008 in mouse brain and stability of these cells during *in vitro* passaging. These data support future development of cell-based therapies using LM-NSC008 cells for various neurodegenerative disorders.

### Migration of LM-NSC008 cells at 6 and 9 months post-injection in naïve mouse brain

To observe changes in the distributions of LM-NSC008 cells in the brain over the months following implantation, we used an orthotopic human NSC xenograft model in which we injected LM-NSC008 cells (5 x 10^5^/4 μl PBS) into the right frontal brain hemispheres of adult male and female NOD scid IL2R gamma null (NSG) mice (n = 10). Mice were euthanized 6 or 9 months after LM-NSC008 injection, and their brains were harvested, fixed, sectioned, and stained for histological examination.

**Fig 2:**
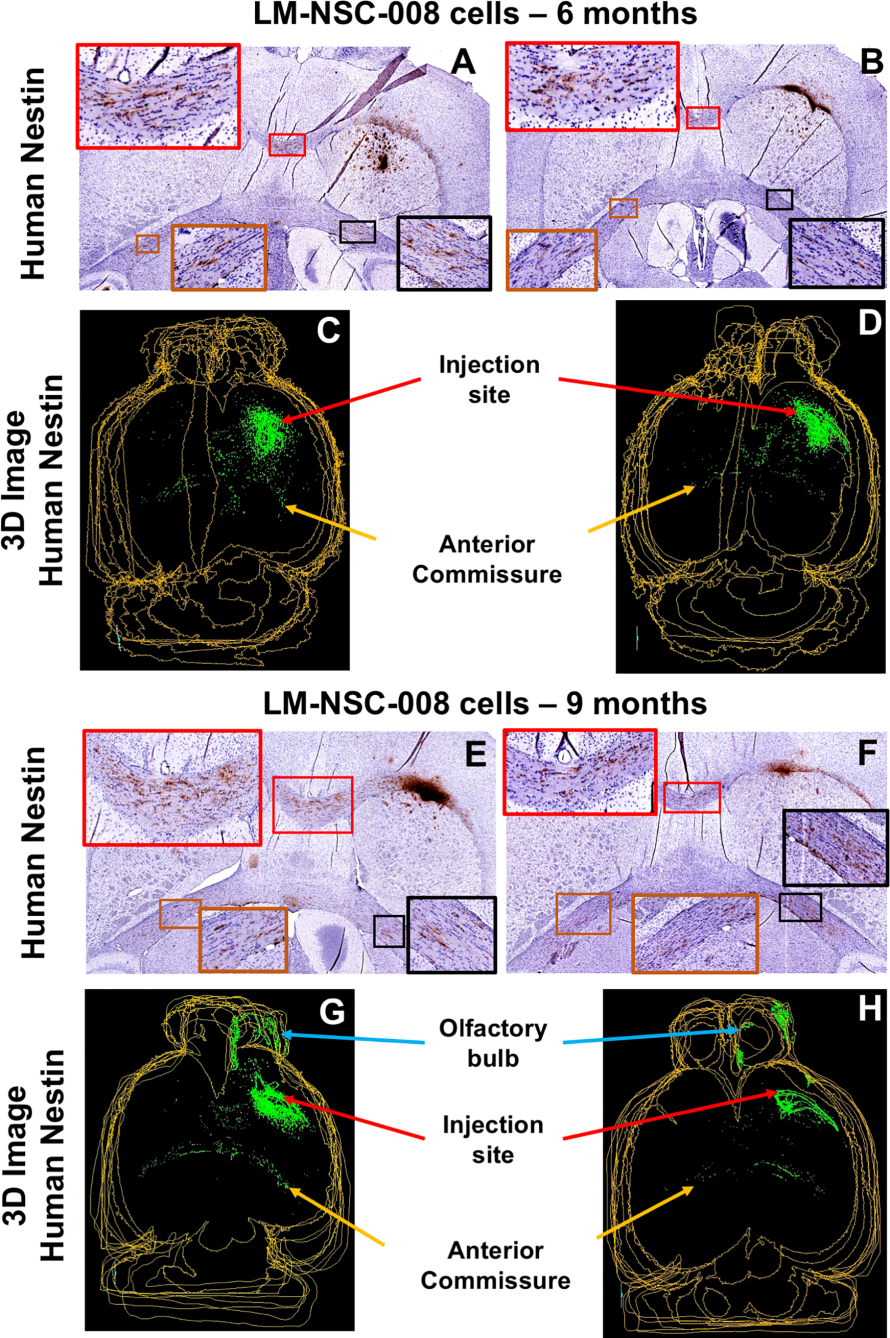
Migration of LM-NSC008 cells at 6 and 9 months post-injection. **(A-D)** IHC staining with hu-nestin antibody of mouse brain sections to identify LM-NSC008 cells (brown) at 6 months **(A, B)** and 9 months **(E, F)** post-injection. **(C, D, G, H)** Three dimensional (3D) reconstruction of mouse brains using Reconstruct software (SynapseWeb, version 1.1). Distribution of LM-NSC008 cells (green) at 6 months **(C, D)** and 9 months **(G, H)** post-injection. Scale bars, 1000 μm.

LM-NSC008 cells were present at the NSC injection site and migrating through the corpus callosum (Fig 2). Accumulation of LM-NSC008 cells was evident in the central corpus callosum by 6 months post-implantation (Fig 2A and 2B), and by 9 months post-implantation an increased number and higher density of NSCs was evident within white matter (WM) distant from the injection site (Fig 2E and 2F), including the anterior commissure (AC). Within the AC, NSCs aggregated along the white matter/grey matter (WM/GM) interfaces (Fig 2). 3D reconstructions of the brain using aligned and Stem123-stained histological sections allowed us to visualize LM-NSC008 cells in 3-dimensions and conduct further analysis using computational methods (Fig 2C, 2D, 2G and 2H). 3D reconstructions and histological sections that were used in computational analyses for all mice are shown in supplemental material (S4–S6 Figs).

### Computational analysis of LM-NSC008 distribution

**Fig 3:**
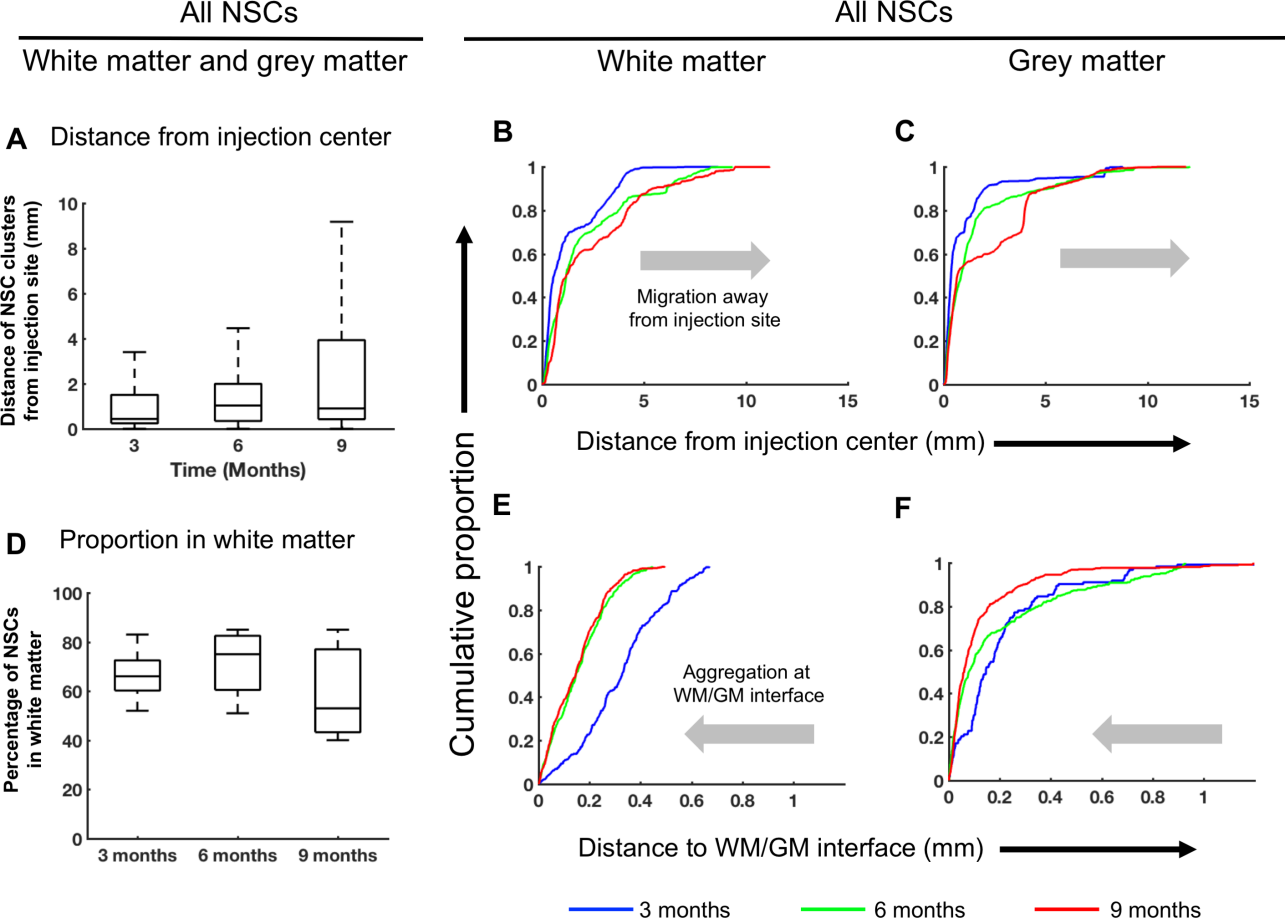
Computational analysis of distribution of LM-NSC008 cells. **(A)** Distances of LM-NSC008 cell clusters from injection sites at 3, 6, and 9 months post-injection. Distances were calculated as Euclidean distances in the 2-dimensional plane. **(B, C)** Cumulative probability distributions of distances of LM-NSC008 clusters from the injection site in WM and GM at 3, 6 and 9 months post-injection. The 9 month curve reflecting a greater distance from the injection center as compared to 3 month curve indicates the migration of NSCs away from the injection site. **(D)** Percentage of LM-NSC008 cells identified in the WM at 3, 6, and 9 months post-injection. **(E, F)** Cumulative probability distribution of distance from white matter/grey matter (WM/GM) interface. The 9 month curve being closer to the WM/GM interface as compared to the 3 month curve indicates that LM-NSC008 cells increasingly aggregated near the WM/GM interface over time.

Migration of LM-NSC008 cells away from the injection site was quantified for three time points: 3 (n = 5), 6 (n = 4) and 9 (n = 3) months post-injection (Fig 3A). To quantify migration of LM-NSC008 cells away from the injection site, we used brain sections stained with hu-nestin and Stem123 antibodies to identify LM-NSC008 cells and lipophilic cyanine dye 1,10-dioctadecyl-3,3,3030-tetramethylindocarbocyanine perchlorate (DiI) to identify myelin associated with white matter tracts. DiI-stained sections were aligned with hu-nestin- or Stem123-stained sections using Reconstruct software (version 1.1.0.1) [23]. Linear distances of LM-NSC008 cells from the injection site (excluding cells within a 1000 pixel [1444 μm] radius from the injection site) as a proportion of the total number of LM-NSC008 cells for each LM-NSC008 cluster was represented as a cumulative probability distribution (CDP). Median distances at 3, 6, and 9 months post-injection were 437 μm, 1030 μm, and 902 μm, respectively. CDPs were also calculated separately for LM-NSC008 cells in white matter (WM; Fig 3B) and grey matter (GM; Fig 3C). We note that NSCs aggregated in the AC ipsilateral to the injection site at the 9 month time point traveled a longer distance than the linear distance measured from the injection site. Roughly 66%, 72%, and 59% of NSCs were found within the WM at 3, 6, and 9 months post-injection, respectively (Fig 3D).

We observed that NSCs aggregated at WM/GM interfaces along the boundary of the AC. To quantify the degree to which NSCs aggregated along the WM/GM interfaces, we calculated the distance between LM-NSC008 cells and the closest WM/GM interface for each brain section and computed the CPDs for the NSCs in both WM (Fig 3E) and GM (Fig 3F). The distances between of NSCs and the nearest WM/GM interface at the 6 and 9-month time points were smaller than those for the 3-month time point, indicating an increased proportion of NSCs closer to WM/GM interfaces. The median distances between NSCs and the nearest WM/GM interface at the 3, 6, and 9 month time points were 263 μm, 118 μm, and 87 μm, respectively (combined WM and GM data).

### Analysis of LM-NSC008 migration and alignment with intrinsic brain structures

**Fig 4:**
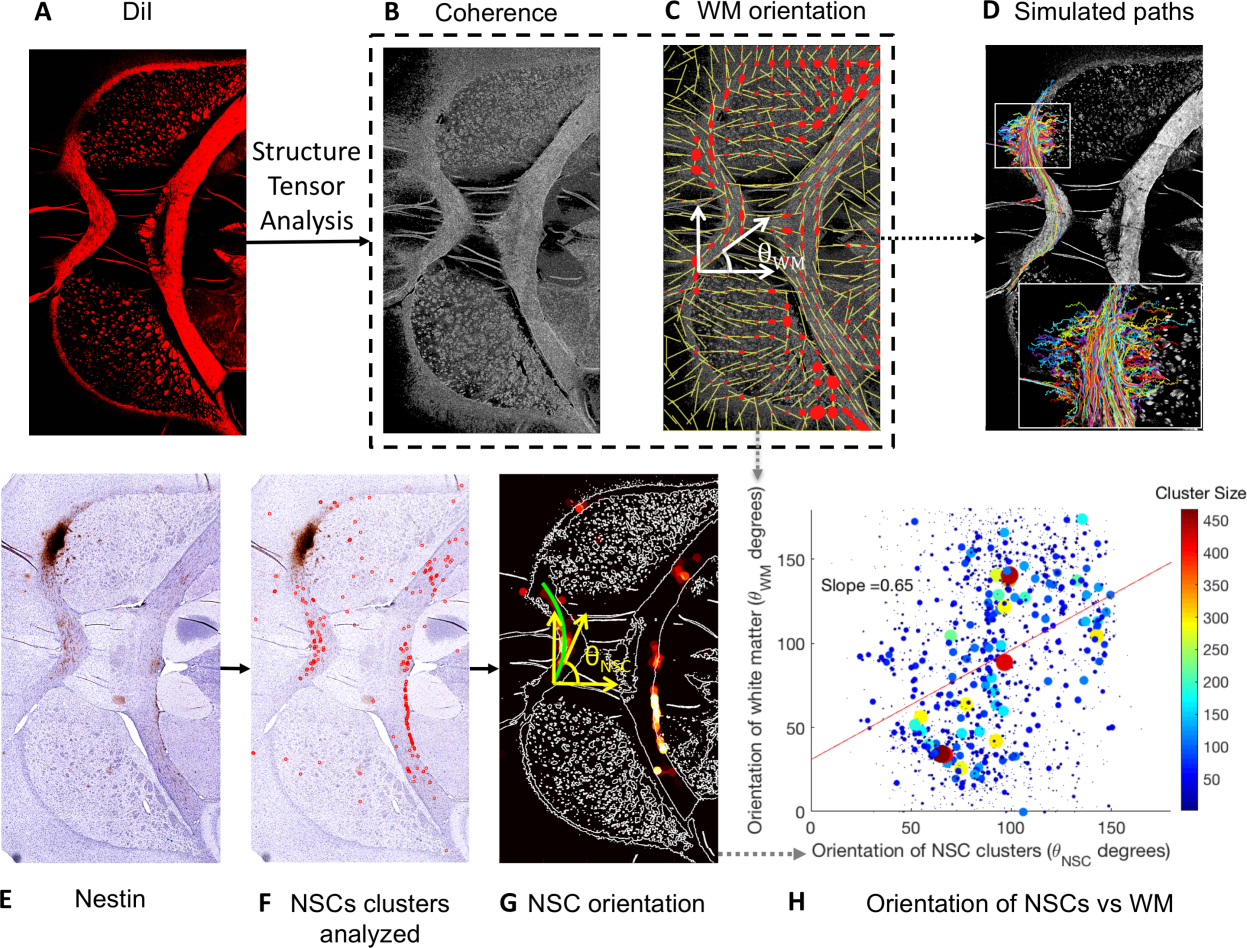
Computational analysis of LM-NSC008 cells and tissue orientation. **(A)** DiI myelin-stained cross-section defining regions of WM. **(B)** The corresponding relative tissue anisotropy (coherence) image generated by the OrientationJ plugin to FIJI with 1 pixel Gaussian kernel. A higher pixel intensity indicates higher anisotropy and vice-versa. **(C)** Orientation vectors of WM overlaid on the coherence image. The angle θ_WM_ is the orientation of the WM. The red dots and the yellow lines indicate the ellipses and the dominant eigenvector calculated via structure tensor analysis (see Supplementary Materials). **(D)** Stochastic simulations of 500 NSC migration paths overlaid on the coherence map evaluated using 5 pixel Gaussian kernel. Preferential migration along the corpus callosum is evident. Inset shows the paths near the seed initialization region (analogous to injection site in a biological experiment) contrasting the directional motility in the WM and GM. **(E)** Nestin-stained cross-section adjacent to the section shown in (A) of naïve mouse brain 9 months after injection of NSCs (brown). **(F)** NSC clusters were identified using color and intensity thresholds. These clusters are dilated and eroded to create a map of NSC density. **(G)** NSC density overlaid on the DiI-defined WM boundaries, excluding the injection site. The green curve through the corpus callosum shows the curve fit through one NSC coalesced region. The angle θ_NSC_ is the orientation angle of an NSC cluster. **(H)** Scatter plot showing the orientation of NSC clusters (θ_NSC_) against the orientation of WM (θ_WM_) weighted by cluster size. The slope of the line indicates the slope of the fit through the scatter plot. Note that the NSCs clusters analyzed above include clusters in both WM and GM. Separate scatter plots for NSCs in WM and GM are shown in the supplemental data.

The alignment of LM-NSC008 cells with intrinsic brain structures was evaluated by correlating the orientation of the LM-NSC008 cells with the orientation of the brain tissue. First, tissue orientation was quantified on a section of DiI-stained tissue (Fig 4A) using structure tensor analysis with the OrientationJ [24] plugin for FIJI (Fig 4B and 4C). The eigenvector associated with the largest eigenvalue of the structure tensor was used to calculate the dominant direction of the tissue at each pixel by calculating the angle of the principle eigenvector with respect to the abscissa axis (θ_WM_) (Fig 4C). NSCs were identified by positive nestin or Stem123 staining (Fig 4E) on a tissue section adjacent to the DiI-stained section. Clusters of NSCs (Fig 4F) were used to determine the orientation of the LM-NSC008 cells with respect to each other (Fig 4G). The orientation of the LM-NSC008 cells (θ_NSC_) was calculated and was found to positively correlate with the orientation of the DiI-defined WM (θ_WM_) (Fig 4H; slope = 0.65, r^2^ = 0.22). The linear regression was weighted by the total number of NSC clusters in each coalesced region analyzed. The distribution of LM-NSC008 cells in the brain combined with the correlation of the orientation of the cells with the intrinsic structures of the brain suggests that NSCs preferentially migrate along WM and align their major axis with the structure of the brain tissue.

Using a computational model, we performed simulations of NSC migration in the brain to study potential NSC migration paths. We initialized 500 simulations at random points within a circular region of the injection site, located near the corpus callosum (Fig 4D). The orientation vectors at each point on the DiI image were calculated using OrientationJ with 5 pixel Gaussian kernel (to calculate stable θ_WM_). These calculated θ_WM_ were used to simulate NSC migration in the WM. In the GM, NSC migration angles were randomly sampled from a uniform distribution of 0–180 degrees relative to the direction of the migration vector of the previous time step. The paths were truncated if the NSC path intersected itself. Thus, we formulated a predictive model of NSC migration in the brain, consistent with observed NSC distributions at the 3, 6, and 9 month post-injection time points. A potential limitation of our approach is that we considered only NSC migration within the brain parenchyma, and did not account for leptomeningeal migration through the cerebrospinal fluid or via intravascular dispersion. Another limitation of the analysis is the two-dimensional analysis of an inherently three-dimensional process.

## Discussion

The LM-NSC008 cell line demonstrated long-term stability and lack of tumorigenicity *in vitro* and *in vivo*. Propagation of LM-NSC008 cells using Quantum Cell Expansion bioreactor (Terumo BCT) demonstrated ease of production in sufficient quantities for clinical trials (S1 Fig). These data support the further development of immortalized LM-NSC008 cells for allogeneic use in the treatment of brain tumors and injury and other neurodegenerative diseases.

In the absence of any pathological injury, LM-NSC008 cells migrated along the corpus callosum (CC) when injected into right frontal lobe. Migrating LM-NSC008 cells localized to the CC (especially the central CC) by 3 months after injection [14]. Analysis of NSC distribution in brains 9 months post-injection showed NSCs aggregated at the interface of the WM/GM in the CC and AC, which was consistent with a nonlinear anisotropic migration predicted by mathematical models of cellular movement with differential rates of migration in the tissue [25].

The brain responds to tumor or injury by activating a variety of defense mechanisms aimed at repairing neuronal damage [26–28]. These mechanisms include enhanced synthesis and secretion of neurotrophins and release of pro- and anti-inflammatory molecules that trigger mobilization and migration of endogenous NSCs to the sites of brain injury. Similar cues are involved in attraction of exogenous NSCs to the site of injury [29]. Three independent processes, determine the success of cell-based therapies: 1) survival of the administered cells, 2) trafficking of the cells to the injured/tumor area; and 3) integration of the cells into the host's neuronal circuits [29, 30]. Because transplanted NSCs can migrate and specifically home into the sites of injury, they have potential therapeutic value in the treatment of diverse neuronal disorders [31]. We developed a method of quantitative analysis of NSC migration that should be useful in analyzing migration of endogenous, exogenous and tumor/injury specific or endogenously cued migration and interpretation of NSC fate in the brain.

The presented computational analysis of NSC migration and distribution provides proof-of-concept for the development of a more mechanistic computational model that can be used to predict NSC migration paths. Although the calculation of tissue orientation (eigenvectors) may appear complicated at first, the task is easily accomplished using the OrientationJ plugin of the open source image analysis software FIJI/ImageJ. Mathematical models of preferential migration along WM tracts have been used to predict distributions of malignant tumor cells, and may also be used to predict NSC migration routes [32–34]. We suggest that quantitative measures of tissue orientation and WM tracts determined from magnetic resonance images [35] can be used in a diffusion tensor imaging tractography-like approach to describe the most likely routes of NSC migration. The presence of chemo-attractants or repulsive signals can easily be incorporated into the computational framework to predict migration rates and routes of NSCs to sites of tumor or TBI. Such a model could greatly enhance the translational potential of delivery of NSCs to treat brain tumors and other brain pathologies by allowing us to identify the most efficient targets for NSC therapy based on the most likely route of migration to the target site. In addition, it could ultimately enable choosing the optimal location for NSC administration to a patient to achieve maximum therapeutic effect.

## Supporting information

S1 Fig Morphology of LM-NSC008 cells in culture and in naïve non-tumor bearing mouse brain. **(A-C)** Images of LM-NSC008 cells in culture at passages 5, 10, and 45. Scale bar, 100 μM. (D) LM-NSC008 cells were plated in 24-well plates at a density of 2 x 10^4^ cells/cm^2^ (40,000 cells/per well). Cells were grown for 10 days and imaged every 12 h, using IncuCyte S3 Live Cell Analysis. Media was changed every 3 days. Experimental data is represented as mean ± SD of 2 independent assays performed in quadruplicate. **(E, F)** Propagation of LM-NSC008s at passage 4 using a Quantum Cell Expansion bioreactor from Terumo BCT. **(E, F)** Cell culture images of LM-NSC008 cells pre- and post-growth in the QCE. **(G)** Expression of biomarkers on LM-NSC008 cells pre- and post-growth in the QCE.
(TIF)

S2 Fig Tissue anisotropy computational analysis. Directed and random motion relationship to tissue structure. Three dimensional representation of the eigenvectors and eigenvalues of the structure tensor that characterizes tissue anisotropy in white **(A)** and grey **(B)** matter. Directed and random migration of NSCs can be explained mathematically by alignment with the principle eigenvector of tissue structure. WM was imaged using DiI **(C)** and MBP **(D)**. Histograms of tissue orientation in regions of the corpus callosum and the anterior commissure are shown for comparison. Comparable WM orientation between the two images is seen.
(TIF)

S3 Fig Sensitivity study of correlation of orientation of NSCs with white matter tracts. Sensitivity study of the orientation of NSCs as a function of the circularity of the region generated in the NSC density map. Inclusion of highly circular regions in the orientation analysis reduced the slope of the regression fit between the NSCs and the white matter tracts. The slope of the regression line was insensitive to selection of regions of interest with circularity greater than 0.7, therefore these coalesced regions were not included in the orientation analysis.
(TIF)

S4 Fig Migration of LM-NSC008 cells at 3 months post-injection. Active migration of NSCs along the corpus callosum was visualized using histological sections stained with human-specific nestin antibodies.
(TIF)

S5 Fig Migration of LM-NSC008 cells at 6 months post-injection. Active migration and localization of NSCs within the corpus callosum and the anterior commissure is shown.
(TIF)

S6 Fig Migration of LM-NSC008 cells at 9 months post-injection. Active migration and localization of NSCs in the corpus callosum, anterior commissure and the olfactory bulb is shown. Increased numbers of NSCs as compared to the 6 month post-injection data are observed. Notably, accumulation of the NSCs at the interface of WM and GM was observed in the anterior commissure.
(TIF)

S7 Fig NSC migration from injection site. Distributions of distances of NSC clusters from the injection site at 3, 6, and 9 months post-injection. Bars represent medians, box limits indicate the first and the third quartiles while the whiskers indicate limits of ± 2.7 times the standard deviation (~ 99.3% coverage) assuming normal distribution. Outliers are shown as crosses.
(TIF)

S8 Fig Temporal dynamics of NSC orientation in white and grey matter. Analysis of NSC orientation with WM over time. Correlation of NSC alignment with the orientation of the WM was greater at **(A)** 3 months than at **(B)** 6 and **(C)** 9 months post-injection. Correlation of NSC alignment with the orientation of GM at **(D)** 3 months, **(E)** 6 months, and **(F)** 9 months. Correlation coefficients in GM were insignificant. θ_WM_ indicates the tissue orientation calculated via OrientationJ in WM and θ_GM_ indicates the tissue orientation in GM.
(TIF)

S1 File Supplemental methods. This supplemental file contains methods regarding Tissue anisotropy computational analysis, Sensitivity study of correlation of orientation of NSC migration with white matter tracts, Analysis of NSC migration from injection site, and Temporal dynamics of NSC orientation in white and grey matter.
(DOCX)
