# Supplementary figures and images for: Long-term stability and computational analysis of migration patterns of L-MYC immortalized neural stem cells in the brain
Source: PLoS One. 2018 Aug 2;13(8):e0199967. doi: 10.1371/journal.pone.0199967 (PMC6071994; doi:10.1371/journal.pone.0199967)

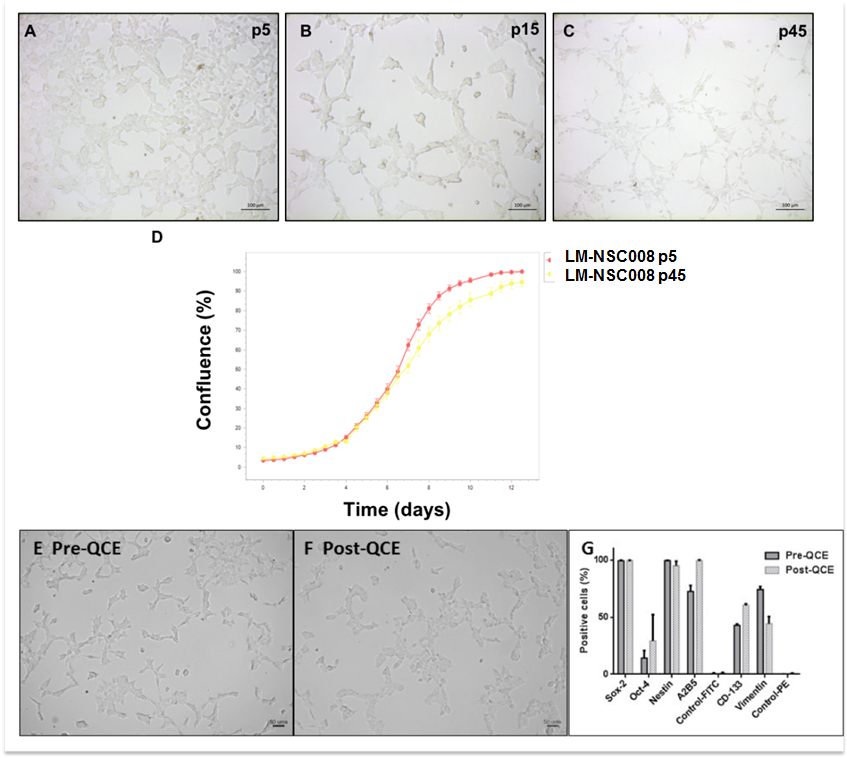

Supplement: S1 Fig — (A-C) Images of LM-NSC008 cells in culture at passages 5, 10, and 45. Scale bar, 100 μM. (D) LM-NSC008 cells were plated in 24-well plates at a density of 2 x 10^4^ cells/cm2 (40,000 cells/per well). Cells were grown for 10 days and imaged every 12 h, using IncuCyte S3 Live Cell Analysis. Media was changed every 3 days. Experimental data is represented as mean ± SD of 2 independent assays performed in quadruplicate. (E, F) Propagation of LM-NSC008s at passage 4 using a Quantum Cell Expansion bioreactor from Terumo BCT. (E, F) Cell culture images of LM-NSC008 cells pre- and post-growth in the QCE. (G) Expression of biomarkers on LM-NSC008 cells pre- and post-growth in the QCE. (TIF) [file pone.0199967.s001.tif]

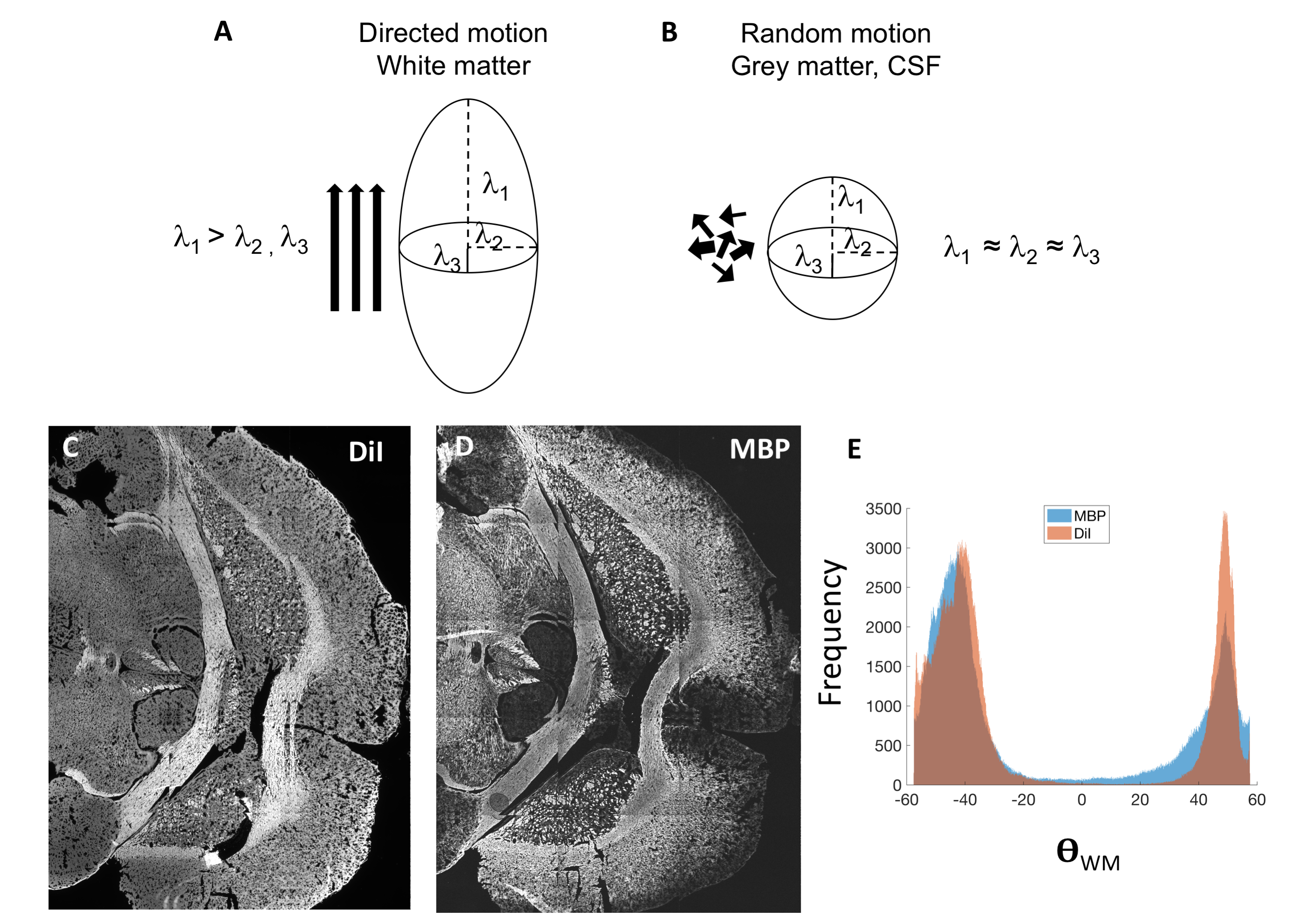

Supplement: S2 Fig — Directed and random motion relationship to tissue structure. Three dimensional representation of the eigenvectors and eigenvalues of the structure tensor that characterizes tissue anisotropy in white (A) and grey (B) matter. Directed and random migration of NSCs can be explained mathematically by alignment with the principle eigenvector of tissue structure. WM was imaged using DiI (C) and MBP (D). Histograms of tissue orientation in regions of the corpus callosum and the anterior commissure are shown for comparison. Comparable WM orientation between the two images is seen. (TIF) [file pone.0199967.s002.tif]

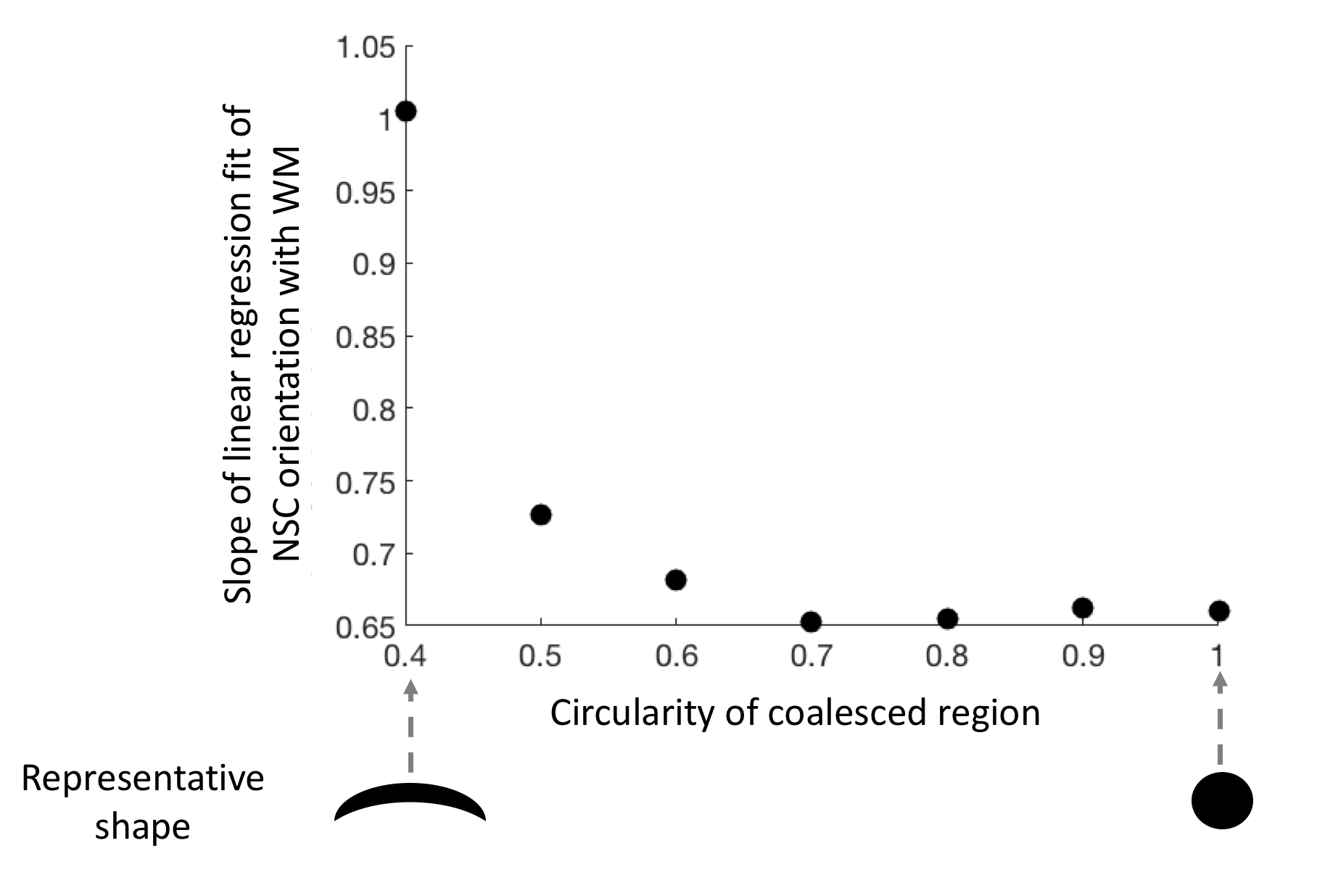

Supplement: S3 Fig — Sensitivity study of the orientation of NSCs as a function of the circularity of the region generated in the NSC density map. Inclusion of highly circular regions in the orientation analysis reduced the slope of the regression fit between the NSCs and the white matter tracts. The slope of the regression line was insensitive to selection of regions of interest with circularity greater than 0.7, therefore these coalesced regions were not included in the orientation analysis. (TIF) [file pone.0199967.s003.tif]

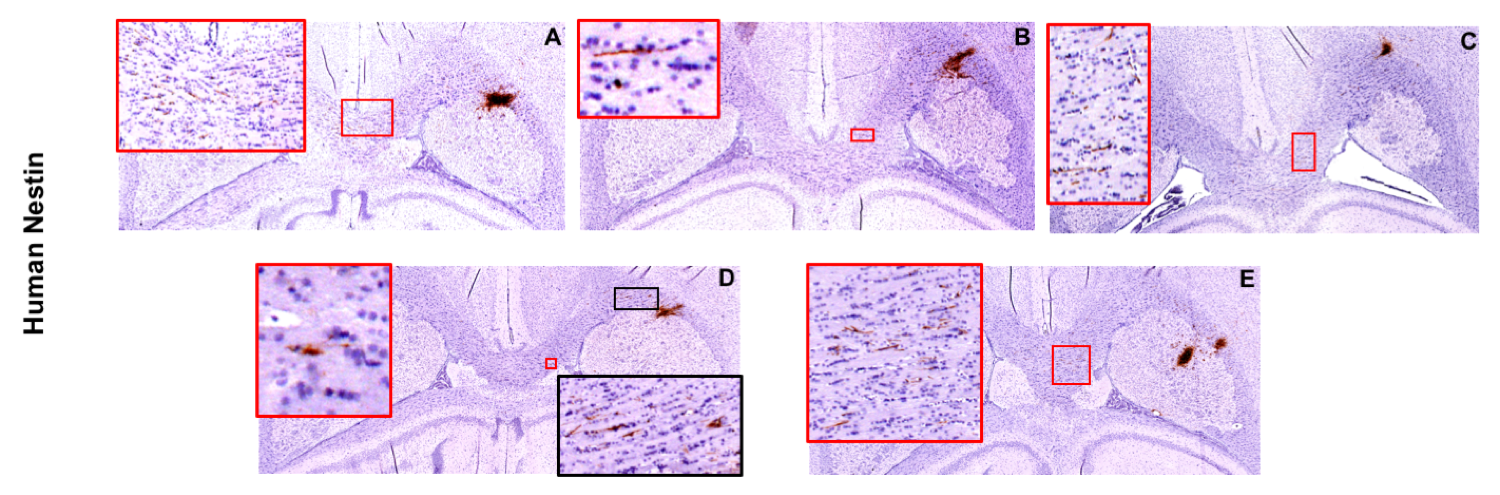

Supplement: S4 Fig — Active migration of NSCs along the corpus callosum was visualized using histological sections stained with human-specific nestin antibodies. (TIF) [file pone.0199967.s004.tif]

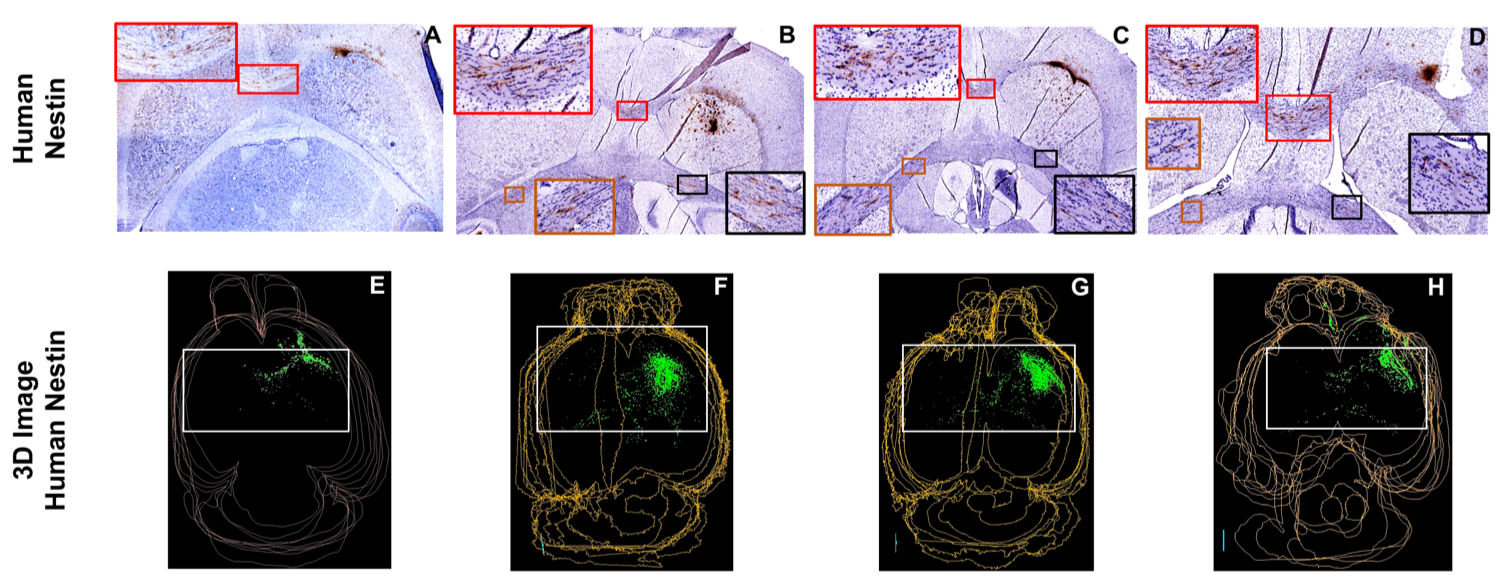

Supplement: S5 Fig — Active migration and localization of NSCs within the corpus callosum and the anterior commissure is shown. (TIF) [file pone.0199967.s005.tif]

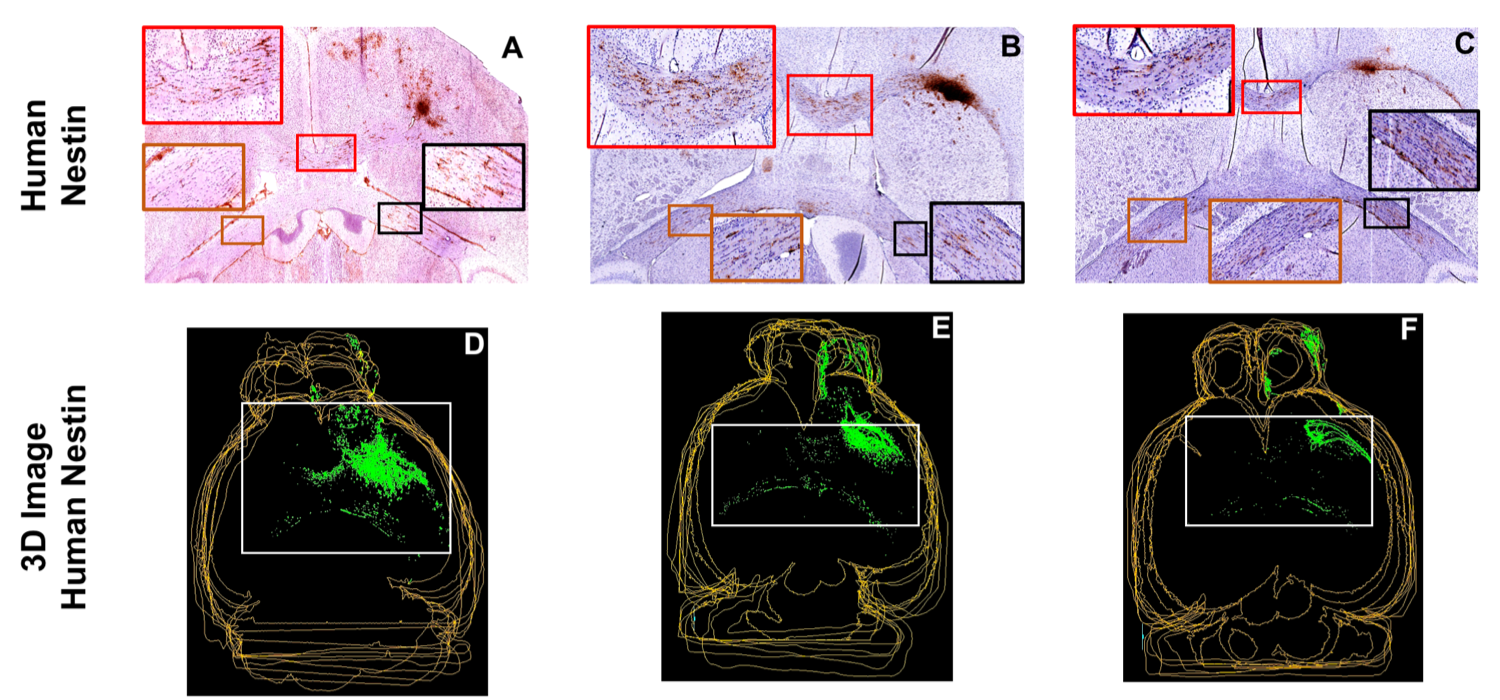

Supplement: S6 Fig — Active migration and localization of NSCs in the corpus callosum, anterior commissure and the olfactory bulb is shown. Increased numbers of NSCs as compared to the 6 month post-injection data are observed. Notably, accumulation of the NSCs at the interface of WM and GM was observed in the anterior commissure. (TIF) [file pone.0199967.s006.tif]

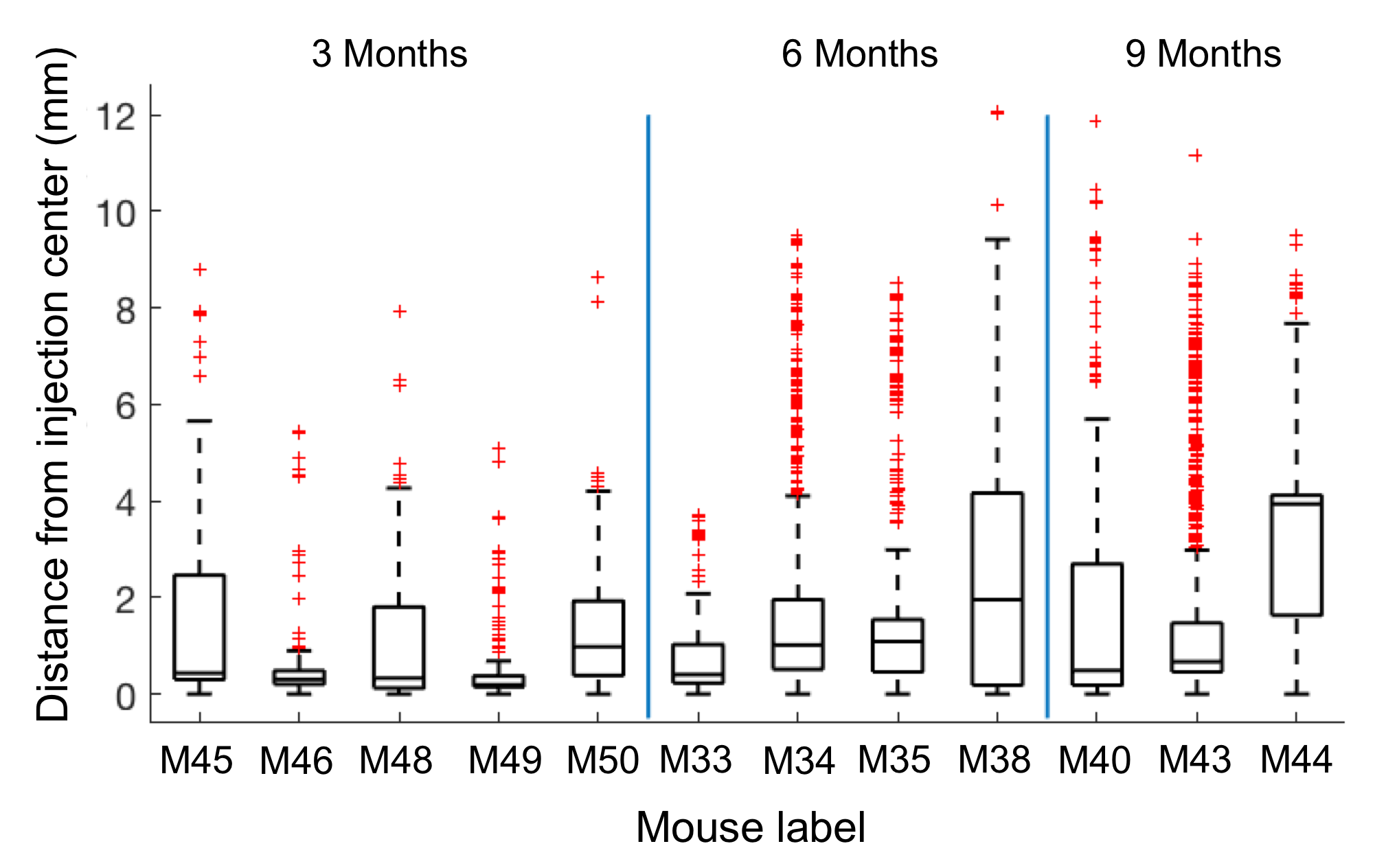

Supplement: S7 Fig — Distributions of distances of NSC clusters from the injection site at 3, 6, and 9 months post-injection. Bars represent medians, box limits indicate the first and the third quartiles while the whiskers indicate limits of ± 2.7 times the standard deviation (~ 99.3% coverage) assuming normal distribution. Outliers are shown as crosses. (TIF) [file pone.0199967.s007.tif]

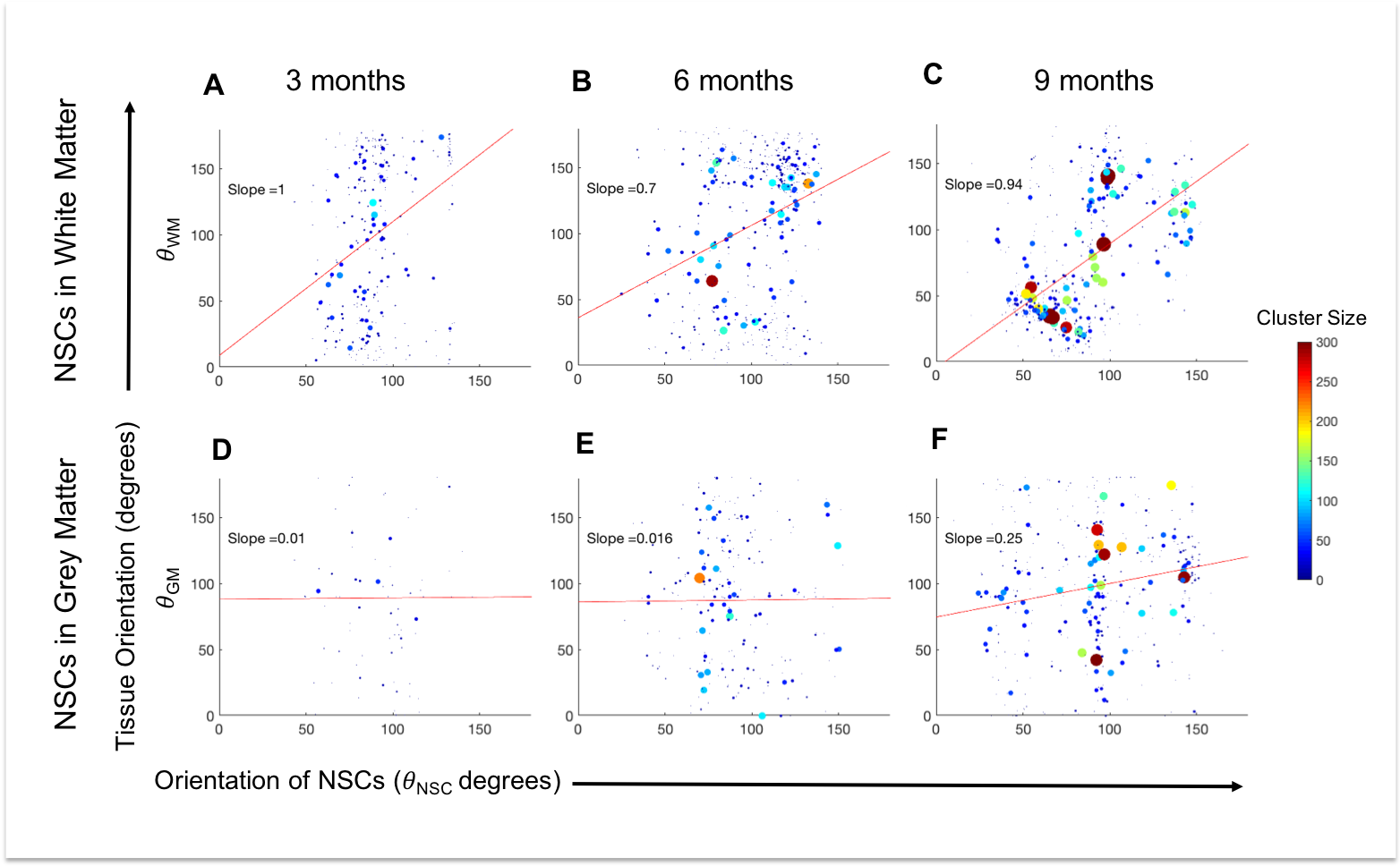

Supplement: S8 Fig — Analysis of NSC orientation with WM over time. Correlation of NSC alignment with the orientation of the WM was greater at (A) 3 months than at (B) 6 and (C) 9 months post-injection. Correlation of NSC alignment with the orientation of GM at (D) 3 months, (E) 6 months, and (F) 9 months. Correlation coefficients in GM were insignificant. θWM indicates the tissue orientation calculated via OrientationJ in WM and θGM indicates the tissue orientation in GM. (TIF) [file pone.0199967.s008.tif]
