# Supplementary material for: Long-term stability and computational analysis of migration patterns of L-MYC immortalized neural stem cells in the brain
Source: PLoS One. 2018 Aug 2;13(8):e0199967. doi: 10.1371/journal.pone.0199967 (PMC6071994; doi:10.1371/journal.pone.0199967)
Supplement: S1 File — This supplemental file contains methods regarding Tissue anisotropy computational analysis, Sensitivity study of correlation of orientation of NSC migration with white matter tracts, Analysis of NSC migration from injection site, and Temporal dynamics of NSC orientation in white and grey matter. (DOCX) [file pone.0199967.s009.docx]

**Supporting Information**

**Tissue anisotropy computational analysis**

White matter (WM) composed of bundles of myelinated axons possesses an inherent orientation based on the direction of these axons and is fundamentally different from grey matter (GM). Structure tensor analysis can be used to quantify the orientation of WM axons. Eigenvectors and their corresponding eigenvalues (λ) of the structure tensor provide direction and relative orientation of the tissue. Cell migration along oriented WM structures as compared to less structured GM is illustrated in 3D (S2 Fig) although analysis in this manuscript is performed in 2D. WM possessing inherent anisotropy generates a dominant eigenvalue. We show the analysis applied to two different WM imaging techniques: using DiI or MBP. Tissue orientation calculated from images of both these techniques in the corpus callosum and anterior commissure are comparable.

**Sensitivity study of correlation of orientation of NSC migration with white matter tracts**

Fig. 4H shows the correlation between the orientation of the NSCs among themselves and the WM tracts. The orientation of the NSC clusters with themselves was calculated first by dilating each NSC cluster center coordinate by 200 pixels and eroding the region created by 100 pixels to generate a coalesced region. Because of the dilation and erosion procedure, a single cell or cluster distant from other NSCs would present itself as a circular region on the NSC density map. Such a cell or cluster is not useful for analyzing the orientation of NSCs among themselves. Therefore, to eliminate such regions, the circularity of each coalesced region created as a result of this dilation/erosion procedure within the NSC density map was calculated using the formula shown in equation S1 below:

$Circularity=\frac{4\pi A}{p^{2}}$ (eq S1)

where A and p are the area and perimeter of the coalesced region, respectively. A region with circularity = 1 indicates a perfect circle with infinite symmetry and therefore no unique orientation. Circularity values of regions having a dominant orientation direction have a value less than one. We performed a sensitivity study of the impact of circularity on the slope of the regression line shown in figure 4H. Increased correlation between the orientation of NSCs with respect to each other and the WM was found with decreasing circularity. A cutoff circularity value of 0.7 was found based on this sensitivity curve (S3 Fig). Coalesced regions having circularity greater than 0.7 were eliminated from the orientation analysis.

**Analysis of NSC migration from injection site**

The distance of each NSC cluster was analyzed as a function of distance from the injection site to elucidate the migration of NSCs with time. Thus, a boxplot of distances of NSC clusters from injection site was created for each mouse (S7 Fig). The distance of NSC clusters from the injection site increased with time, as also demonstrated in Fig. 3A. It should be noted that, in brains from mice euthanized 9 months after NSC injection, the NSC clusters in the ipsilateral side of the AC as the injection site contributed to distances less than the actual path traversed.

**Temporal dynamics of NSC orientation in white and grey matter**

Because a correlation of NSC orientation with WM was found (Fig. 4F), the temporal dynamics of this observation at 3, 6, and 9 months after NSC injection were evaluated individually for WM and GM separately. NSC migration direction was highly correlated with the WM as compared to the GM (S8 Fig). NSC orientation with the WM orientation was greatest at 3 months after injection, decreased at 6 months post-injection, and later increased at 9 months post-injection (S8A-C Fig). The slopes of the regression line in the WM at 3, 6, and 9 months post-injection were 1, 0.7, and 0.94 respectively. The values of these slopes of the regression for WM and GM combined at 3, 6, and 9 months were 0.83, 0.50, and 0.68. These results indicate that NSCs travel preferentially along the WM to their final locations within the brain parenchyma.


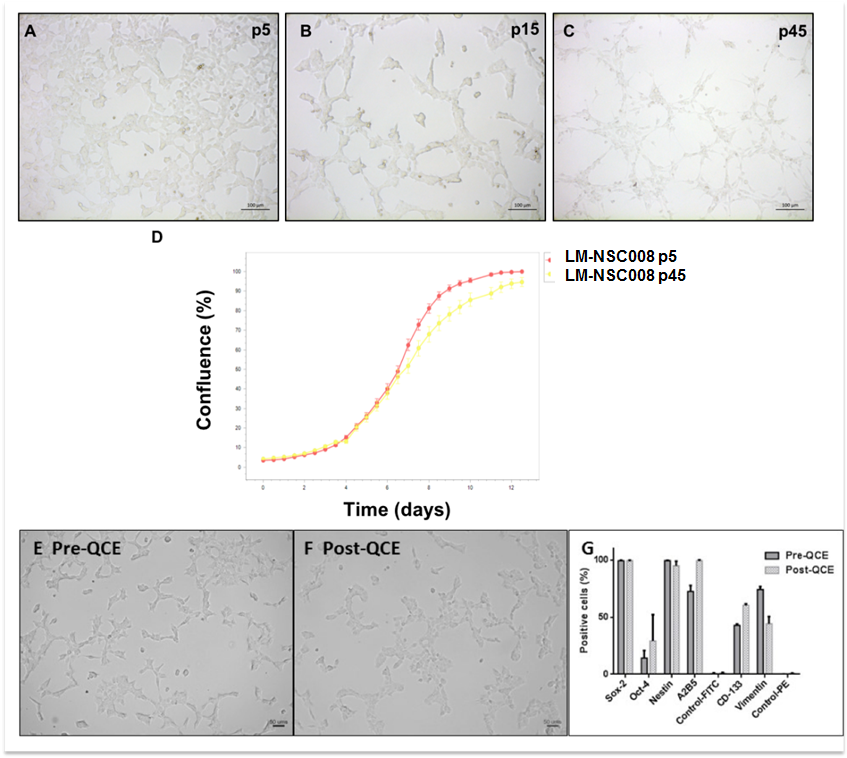


**S1 Fig.** **Morphology of LM-NSC008 cells in culture and in naïve non-tumor bearing mouse brain.** **(A-C)** Images of LM-NSC008 cells in culture at passages 5, 10, and 45. Scale bar, 100 µM. (D) LM-NSC008 cells were plated in 24-well plates at a density of 2 x 10^4^ cells/cm^2^ (40,000 cells/per well). Cells were grown for 10 days and imaged every 12 h, using IncuCyte S3 Live Cell Analysis. Media was changed every 3 days. Experimental data is represented as mean + SD of 2 independent assays performed in quadruplicate. **(E, F)** Propagation of LM-NSC008s at passage 4 using a Quantum Cell Expansion bioreactor from Terumo BCT**. (E, F)** Cell culture images of LM-NSC008 cells pre- and post-growth in the QCE. **(G)** Expression of biomarkers on LM-NSC008 cells pre- and post-growth in the QCE.

**
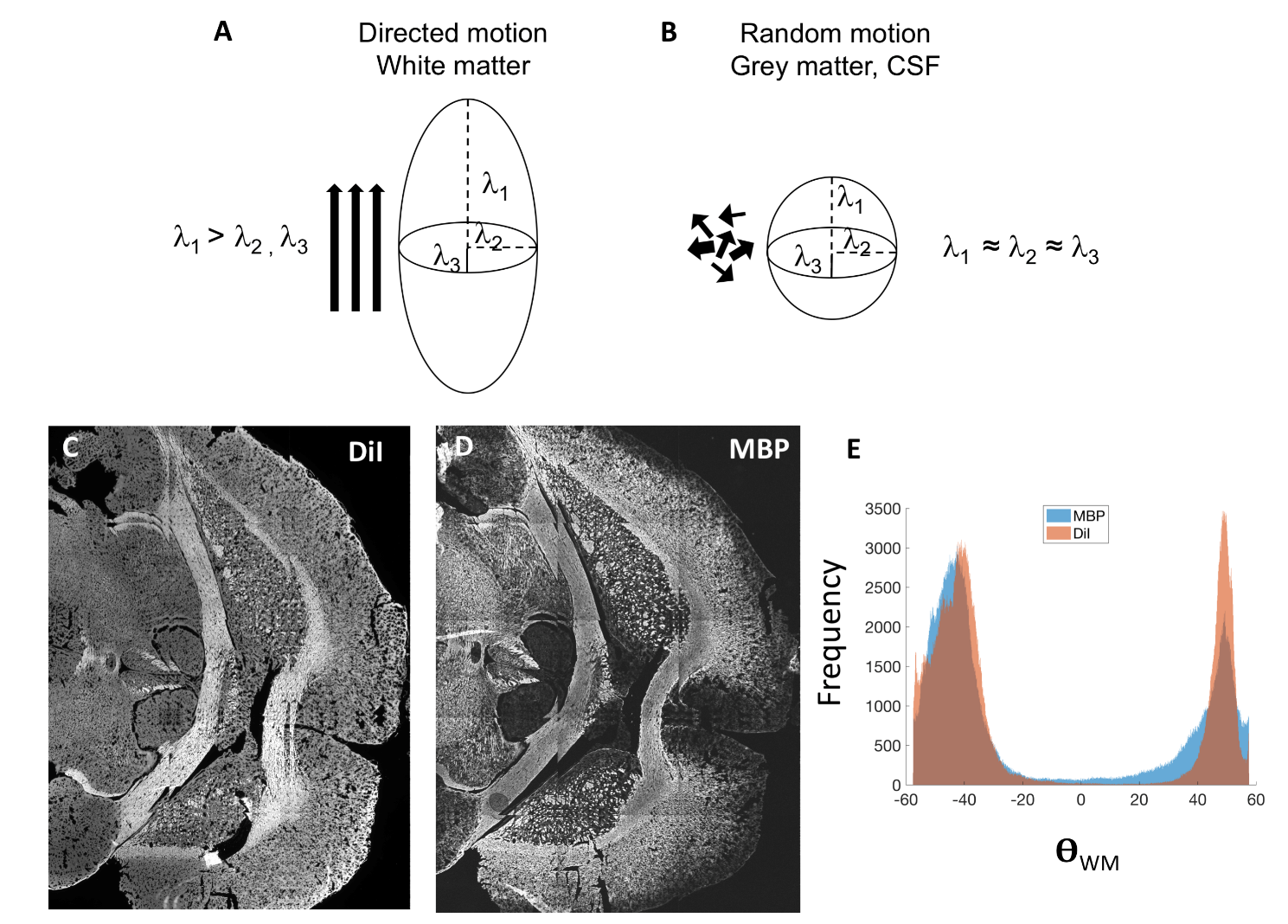
**

**S2 Fig.** **Tissue anisotropy computational analysis**. Directed and random motion relationship to tissue structure. Three dimensional representation of the eigenvectors and eigenvalues of the structure tensor that characterizes tissue anisotropy in white **(A)** and grey **(B)** matter. Directed and random migration of NSCs can be explained mathematically by alignment with the principle eigenvector of tissue structure. WM was imaged using DiI **(C)** and MBP **(D)**. Histograms of tissue orientation in regions of the corpus callosum and the anterior commissure are shown for comparison. Comparable WM orientation between the two images is seen.

**
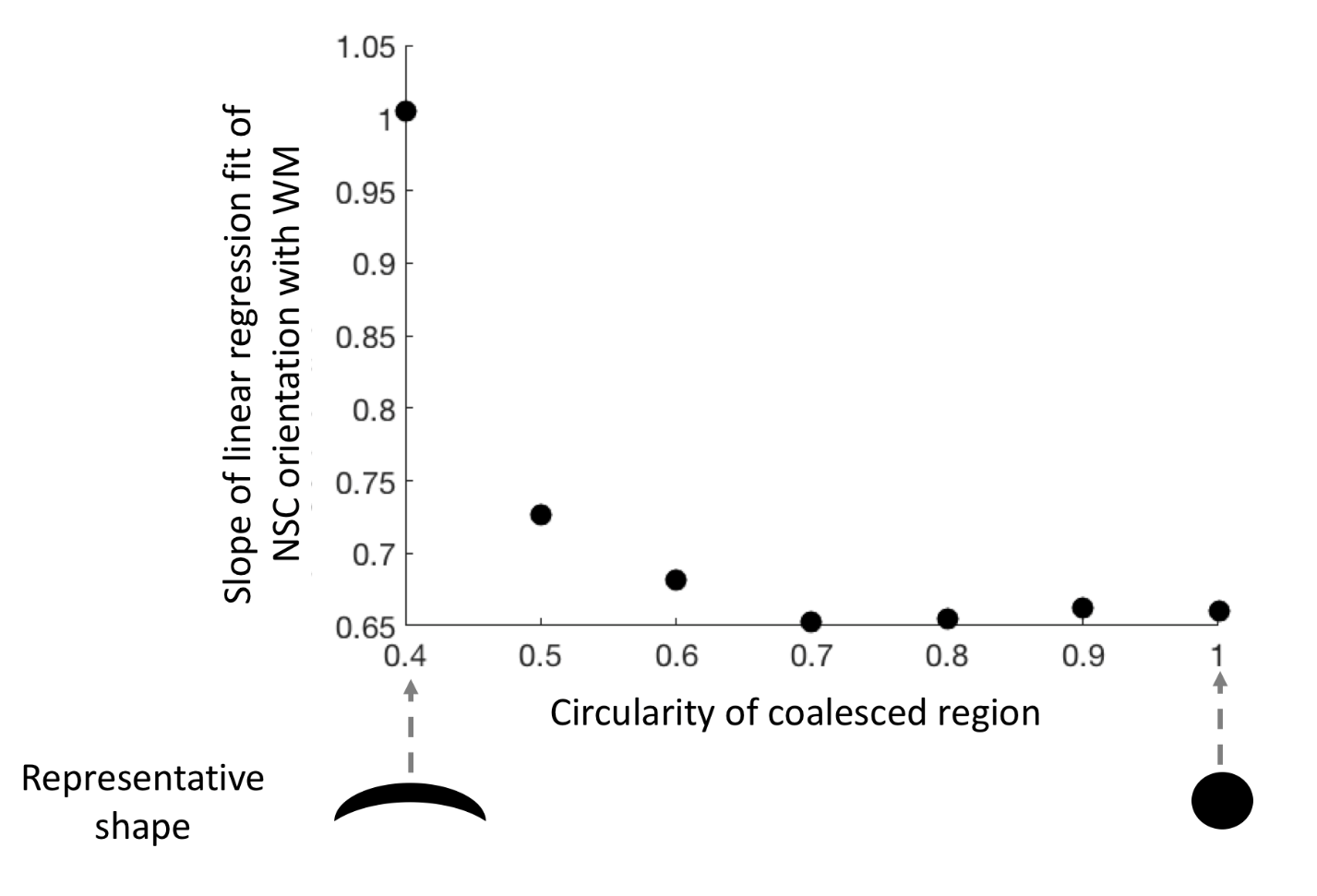
**

**S3 Fig. Sensitivity study of correlation of orientation of NSCs with white matter tracts.** Sensitivity study of the orientation of NSCs as a function of the circularity of the region generated in the NSC density map. Inclusion of highly circular regions in the orientation analysis reduced the slope of the regression fit between the NSCs and the white matter tracts. The slope of the regression line was insensitive to selection of regions of interest with circularity greater than 0.7, therefore these coalesced regions were not included in the orientation analysis.

**
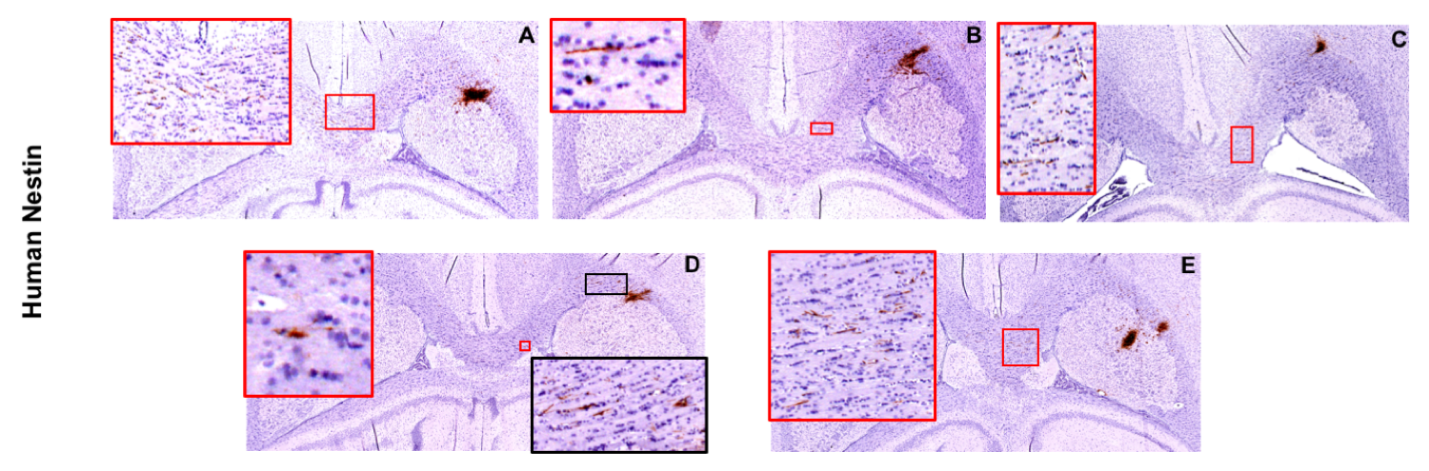
**

**S4 Fig. Migration of LM-NSC008 cells at 3 months post-injection**. Active migration of NSCs along the corpus callosum was visualized using histological sections stained with human-specific nestin antibodies.

**
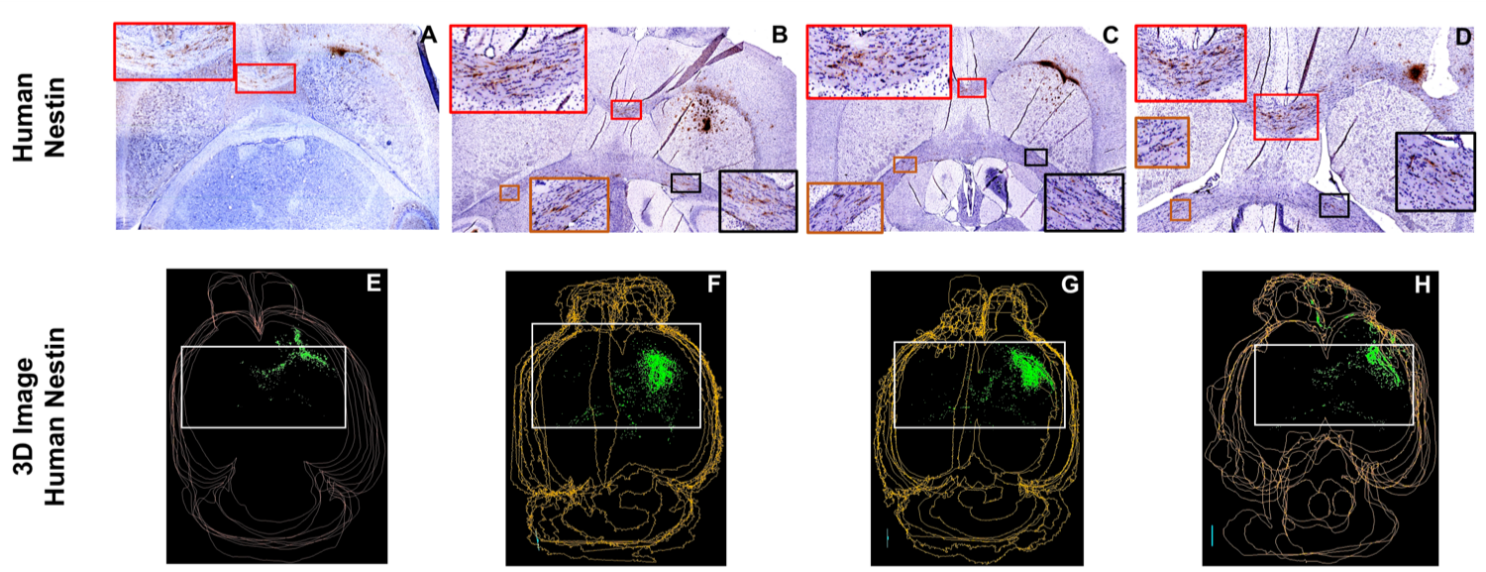
S5 Fig. Migration of LM-NSC008 cells at 6 months post-injection**. Active migration and localization of NSCs within the corpus callosum and the anterior commissure is shown.

**
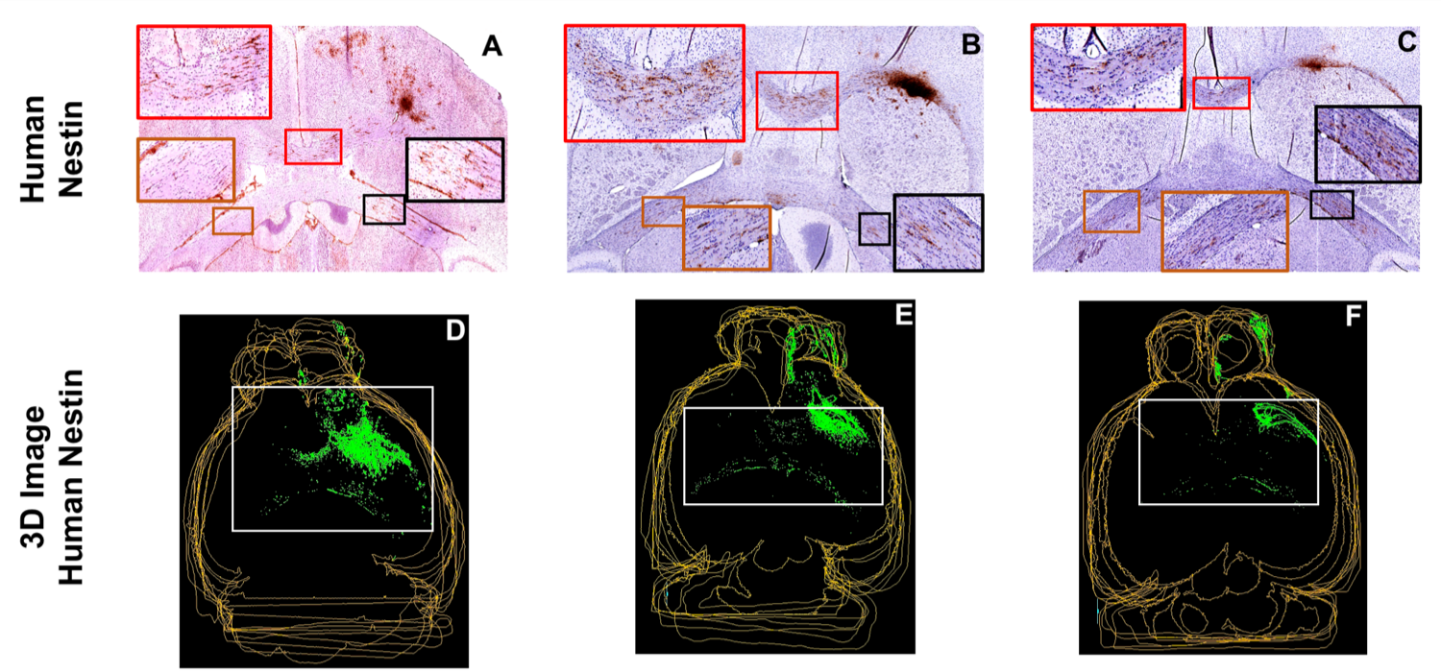
**

**S6 Fig. Migration of LM-NSC008 cells at 9 months post-injection.** Active migration and localization of NSCs in the corpus callosum, anterior commissure and the olfactory bulb is shown. Increased numbers of NSCs as compared to the 6 month post-injection data are observed. Notably, accumulation of the NSCs at the interface of WM and GM was observed in the anterior commissure.


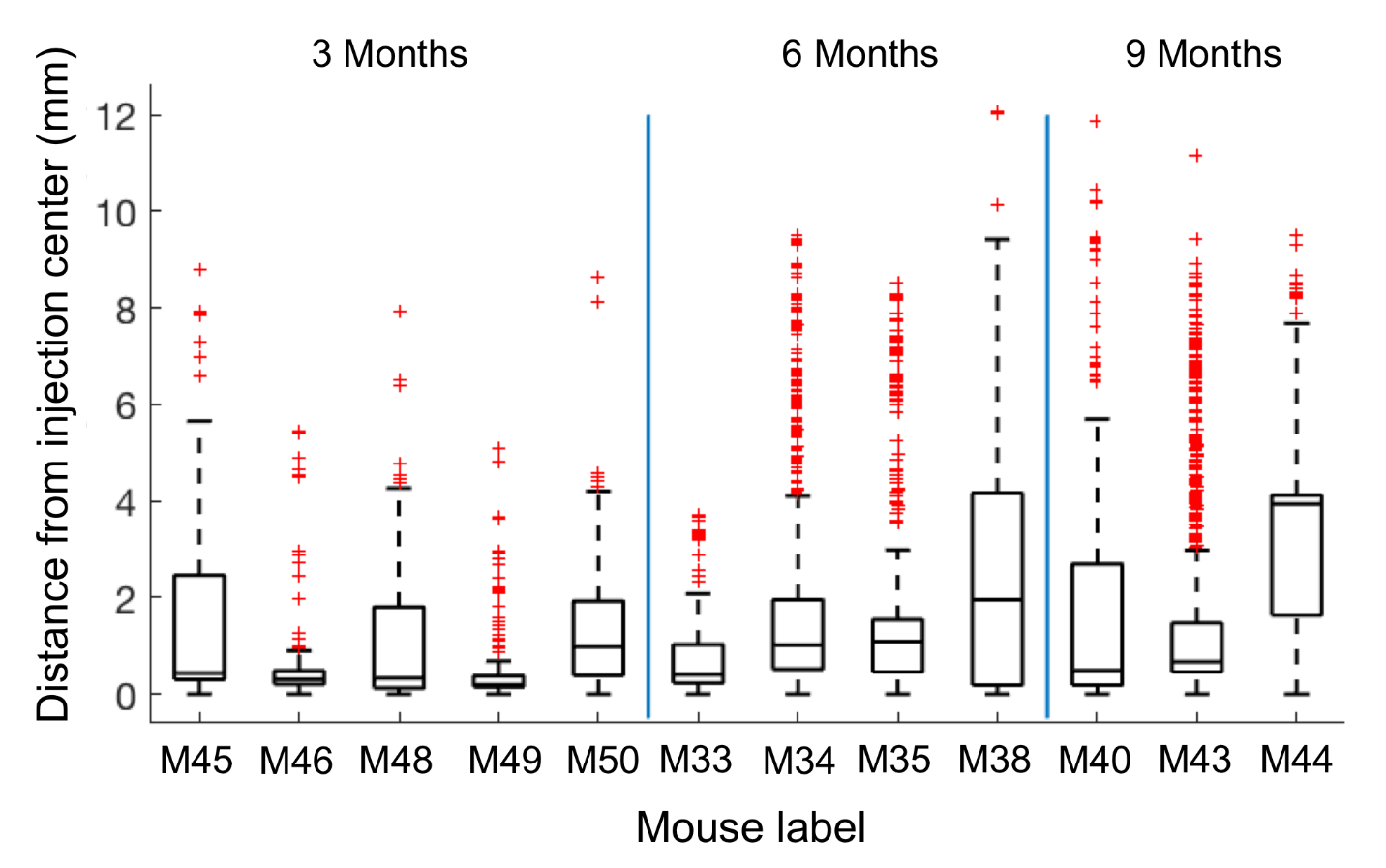


**S7 Fig. NSC migration from injection site.** Distributions of distances of NSC clusters from the injection site at 3, 6, and 9 months post-injection. Bars represent medians, box limits indicate the first and the third quartiles while the whiskers indicate limits of ± 2.7 times the standard deviation (~ 99.3% coverage) assuming normal distribution. Outliers are shown as crosses.


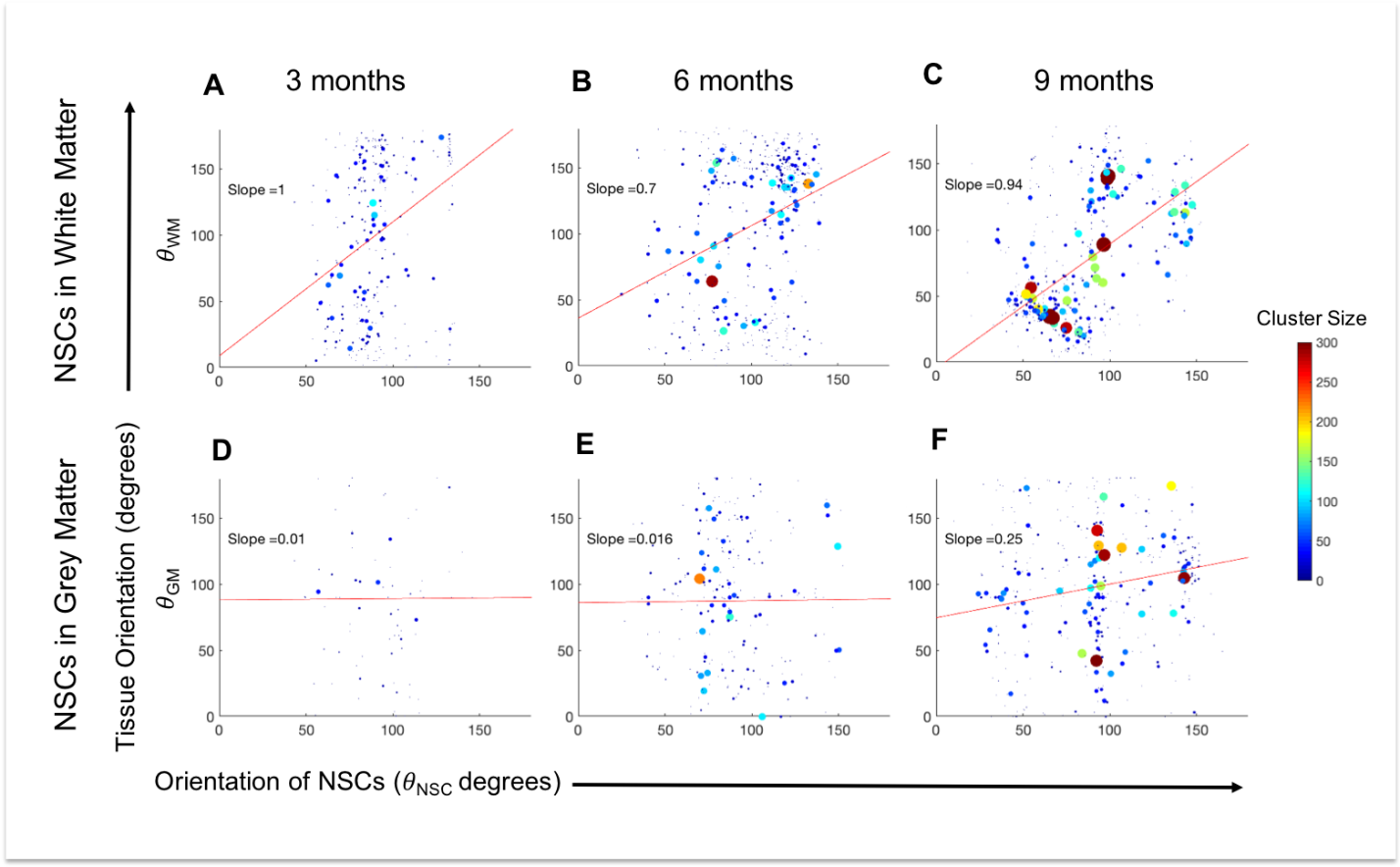


**S8 Fig.** **Temporal dynamics of NSC orientation in white and grey matter.** Analysis of NSC orientation with WM over time. Correlation of NSC alignment with the orientation of the WM was greater at **(A)** 3 months than at **(B)** 6 and **(C)** 9 months post-injection. Correlation of NSC alignment with the orientation of GM at **(D)** 3 months, **(E)** 6 months, and **(F)** 9 months. Correlation coefficients in GM were insignificant. θ_WM_ indicates the tissue orientation calculated via OrientationJ in WM and θ_GM_ indicates the tissue orientation in GM.
